# Rubicon promotes rather than restricts murine lupus and is not required for LC3-associated phagocytosis

**DOI:** 10.1172/jci.insight.155537

**Published:** 2022-04-08

**Authors:** Rachael A. Gordon, Christina Giannouli, Chirag Raparia, Sheldon I. Bastacky, Anthony Marinov, William Hawse, Richard Cattley, Jeremy S. Tilstra, Allison M. Campbell, Kevin M. Nickerson, Anne Davidson, Mark J. Shlomchik

**Affiliations:** 1 Department of Immunology and; 2 Department of Medicine, University of Pittsburgh School of Medicine, Pittsburgh, Pennsylvania, USA.; 3 Institute of Molecular Medicine, Feinstein Institutes for Medical Research, Manhasset, New York, USA.; 4 Department of Pathology, University of Pittsburgh School of Medicine, Pittsburgh, Pennsylvania, USA.; 5 Department of Immunobiology, Yale University School of Medicine, New Haven Connecticut, USA.

**Keywords:** Autoimmunity, Autoimmune diseases

## Abstract

NADPH oxidase deficiency exacerbates lupus in murine models and patients, but the mechanisms remain unknown. It is hypothesized that NADPH oxidase suppresses autoimmunity by facilitating dead cell clearance via LC3-associated phagocytosis (LAP). The absence of LAP reportedly causes an autoinflammatory syndrome in aged, nonautoimmune mice. Prior work implicated cytochrome b-245, β polypeptide (CYBB), a component of the NADPH oxidase complex, and the RUN and cysteine-rich domain-containing Beclin 1–interacting protein (RUBICON) as requisite for LAP. To test the hypothesis that NADPH oxidase deficiency exacerbates lupus via a defect in LAP, we deleted *Rubicon* in the B6.Sle1.*Yaa* and MRL.Fas*^lpr^* lupus mouse models. Under this hypothesis, RUBICON deficiency should phenocopy NADPH oxidase deficiency, as both work in the same pathway. However, we observed the opposite — RUBICON deficiency resulted in reduced mortality, renal disease, and autoantibody titers to RNA-associated autoantigens. Given that our data contradict the published role for LAP in autoimmunity, we assessed whether CYBB and RUBICON are requisite for LAP. We found that LAP is not dependent on either of these 2 pathways. To our knowledge, our data reveal RUBICON as a novel regulator of SLE, possibly by a B cell–intrinsic mechanism, but do not support a role for LAP in lupus.

## Introduction

Systemic lupus erythematosus (SLE) is a multisystem autoimmune disease characterized by loss of tolerance, rampant immune activation, and end-organ damage (1). Loss of tolerance to nuclear antigens and the formation of autoantibodies to nucleic acids and nucleoproteins are hallmarks of SLE pathogenesis. While the sources of autoantigens in lupus remain enigmatic, a failure to adequately dispose of dead cells and resulting debris by macrophages is a leading possibility (2). Indeed, macrophages from a subset of lupus patients exhibit an impaired ability to phagocytose, a finding also observed in murine models (3–6). Moreover, there are several lines of evidence that link dead cell clearance pathways to the development of systemic autoimmunity. Loss of function of complement component 1q (C1q), T cell immunoglobulin and mucin domain-containing 4 (TIM4), and milk fat globule-EGF factor 8 (MFG-E8) result in lupus-like syndromes in humans and in mice (7–13). Taken together, these studies emphasize that inadequate clearance of dead cells can result in an immune response to self and subsequent end-organ damage.

The NADPH oxidase complex, a group of transmembrane and cytosolic enzymes responsible for the respiratory burst critical for microbial killing (14–16), is important for the clearance and degradation of dead cells by macrophages (17–22). Loss-of-function mutations in essential components of the NADPH oxidase including cytochrome b-245, α polypeptide CYBA, cytochrome b-245, β polypeptide (CYBB), and neutrophil cytosolic factor (NCF) 1 and 2 result in chronic granulomatous disease (CGD). A link between CGD and systemic autoimmunity is well established. Male patients with X-linked CGD, characterized by loss-of-function mutations in *CYBB*, are at greater risk of developing a lupus-like disease (23, 24). Moreover, carrier mothers of affected males are more likely to develop SLE, indicating that heterozygous dosing of the *CYBB* allele is sufficient to drive lupus (25, 26). Positional cloning of the Pia4 quantitative trait locus identified a loss-of-function polymorphism in *Ncf1* that was associated with increased arthritis severity in rat models, a finding that was also observed in mice with loss-of-function mutations in *Ncf1* (27, 28). Subsequently, loss-of-function polymorphisms in *NCF1* and *NCF2* were found to confer increased SLE susceptibility across multiple ethnicities (29–32). Over the past 2 decades, multiple mouse models of CGD mirrored increased autoimmunity susceptibility observed in humans (28, 33–38). Collectively, these studies show that the NADPH oxidase complex is critical for the regulation of autoimmune pathology in mice and humans.

Although the mechanism by which NADPH oxidase regulates the anti–self-response is unknown, a compelling hypothesis is that it suppresses autoimmunity by promoting dead cell clearance by myeloid cells (17–20, 39). Recently, LC3-associated phagocytosis (LAP), a process that partially overlaps with autophagy (22, 40, 41), has been implicated in the noninflammatory degradation of dead cell debris by macrophages (41). LAP occurs when certain types of phagocytosed particles that can stimulate aspects of innate immunity cause the recruitment of autophagy machinery to the phagosome, facilitating maturation and the degradation of the engulfed contents (22, 40, 41). Genetic dissection of LAP identified LAP-specific components, e.g., *Cybb* and the RUN and cysteine-rich domain-containing Beclin 1–interacting protein (*Rubicon*) as well as genes required for both LAP and autophagy, e.g., *Beclin1*, Autophagy-related gene 5 (*Atg5*) and *Atg7* (22). Martinez and colleagues reported that genetic deletion of components implicated in LAP only (*Rubicon* and *Cybb*) or in both LAP and canonical autophagy (*Beclin1*, *Atg5*, and *Atg7*) led to an autoinflammatory syndrome in aged, nonautoimmune C57BL/6 mice. These mice developed low-titer autoantibodies and mild renal disease (38). Strikingly, mice deficient in components implicated in canonical autophagy only, i.e., Unc-51–like kinase 1 (ULK1) and FAK family kinase-interacting protein of 200 kDa (FIP200), did not develop systemic autoimmunity with age, suggesting that LAP but not canonical autophagy is required to prevent autoimmunity (38). Interestingly, increased levels of proinflammatory cytokines were present in the serum of these LAP-deficient mice, but antiinflammatory cytokines, such as IL-10, were reduced (38). The authors postulated that in the absence of LAP, dead cells are not cleared in an immunologically silent way and that the inability to generate IL-10 downstream of LAP is a driver of the disease phenotype (38).

While evidence suggests that blocking LAP can drive an anti–self-response in a nonautoimmune setting, the role of LAP and the genes that promote it remain unclear in the context of clinical SLE. We previously showed that deletion of *Cybb* in lupus-prone MRL.Fas*^lpr^* mice led to markedly exacerbated disease (34). As NADPH oxidase is required for LAP, it is possible that the exacerbated disease observed in the context of *Cybb* deficiency is due to inhibition of LAP. If *Cybb* deficiency exacerbates SLE by prevention of dead cell clearance due to a defect in LAP and subsequent inhibition of antiinflammatory cytokine production, then deleting another LAP-specific gene should have a similar phenotype of exacerbated disease in lupus-prone murine models.

We addressed this by genetically deleting another requisite component for LAP, *Rubicon,* in both the MRL.Fas*^lpr^* and B6.Sle1.*Yaa* lupus models and studied the effects on disease in both *Cybb*-deficient and WT genetic backgrounds. We chose the MRL.Fas*^lpr^* model as it is a leading spontaneous, polygenic system to study SLE, recapitulating nearly all features of the human disease (42). Additionally, it has been used to study the role of *Cybb* deficiency in SLE, allowing for a direct comparison of results. Furthermore, studies in MRL.Fas*^lpr^* mice have accurately predicted responses in human translational studies, validating the use of this model in preclinical investigation (42–48). The *Sle1* locus is on the C57BL/6 genetic background and includes lupus susceptibility polymorphisms involving Slam family members (49–52). Combining this locus with the *Yaa* locus in males confers an extra copy of *Tlr7* that drives additional autoreactivity to RNA antigens, resulting in a lupus-like disease characterized by nephritis and early mortality (53).

We found that *Rubicon*-deficient SLE-prone mice did not phenocopy exacerbated lupus observed in *Cybb*-KO mice. In fact, the absence of RUBICON conferred a survival advantage in SLE-prone mice, including *Cybb*-deficient MRL.Fas*^lpr^* mice, and protected them from renal disease. Since our results did not support the published role for LAP in autoimmunity, and in fact showed opposite roles for 2 proteins that are both thought to be critical for LAP, we reassessed whether CYBB and RUBICON are indeed requisite for LAP in macrophages. Surprisingly, we show that LAP is, in fact, dependent on neither of these 2 proteins. Taken together, our data do not support a role for LAP in lupus. Most importantly, to our knowledge, these data highlight RUBICON as a novel regulator of SLE pathogenesis.

## Results

### Rubicon deficiency does not reduce survival in murine SLE.

To determine whether *Cybb* deficiency exacerbates SLE by prevention of dead cell clearance due to a defect in LAP, we genetically deleted another requisite component of LAP, *Rubicon*, in the B6.Sle1.*Yaa* and MRL.Fas*^lpr^* lupus models. *Rubicon* was genetically deleted directly on the MRL.Fas*^lpr^* background using CRISPR/Cas9 (Supplemental Figure 1, A and B; supplemental material available online with this article; https://doi.org/10.1172/jci.insight.155537DS1). To determine whether CYBB and RUBICON act in the same pathway (i.e., LAP), we analyzed *Cybb* and *Rubicon* single and double KO mice in the setting of lupus. SLE pathology was analyzed at 16–18 weeks of age in MRL.Fas*^lpr^* mice and at 8–21 months of age in the B6.Sle1.*Yaa* model unless otherwise indicated.

**Figure 1:**
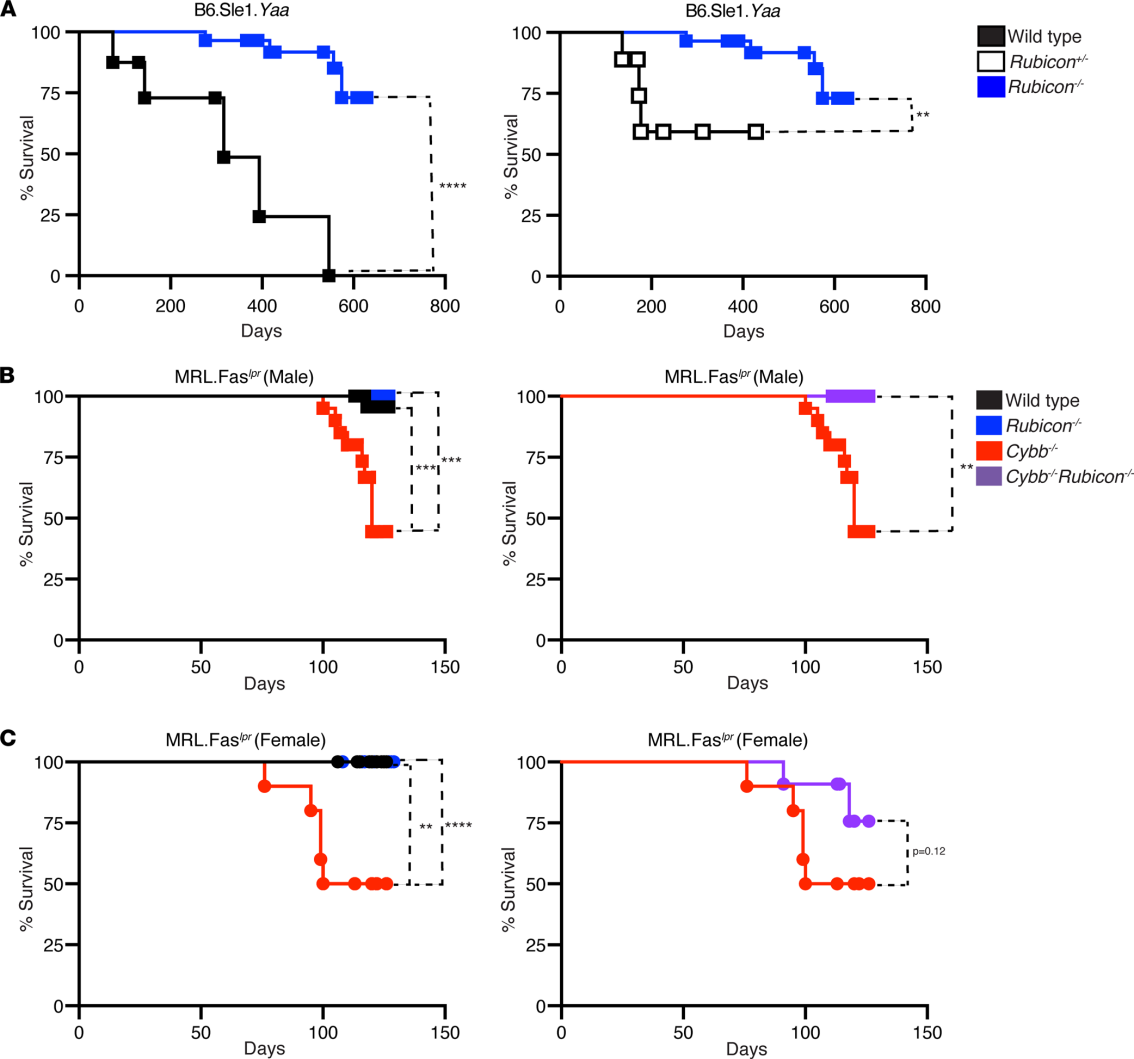
The absence of RUBICON confers a survival advantage in SLE-prone mice. Kaplan-Meier survival curves for (**A**) B6.Sle1.*Yaa*, (**B**) male MRL.Fas*^lpr^*, and (**C**) female MRL.Fas*^lpr^* SLE mice of indicated genotypes. A log-rank test was used to determine statistical significance between Kaplan-Meier curves (**P < 0.01, ***P < 0.001, ****P < 0.0001; and B6.Sle1.*Yaa* WT males *n* = 8; B6.Sle1.*Yaa* *Rubicon^–/+^* males *n* = 9; B6.Sle1.*Yaa* *Rubicon^–/–^* males *n* = 28; MRL.Fas*^lpr^* WT males *n* = 28; MRL.Fas*^lpr^* WT females *n* = 16; MRL.Fas*^lpr^* *Rubicon^–/–^* males *n* = 29; MRL.Fas*^lpr^* *Rubicon^–/–^* females *n* = 27; MRL.Fas*^lpr^* *Cybb^–/y^* males *n* = 20; MRL.Fas*^lpr^* *Cybb^–/–^* females *n* = 10; MRL.Fas*^lpr^* *Rubicon^–/–^Cybb^–/y^* males *n* = 16; and MRL.Fas*^lpr^* *Rubicon^–/–^Cybb^–/–^* females *n* = 11 mice per group).

Surprisingly, *Rubicon* deficiency actually protected from, rather than exacerbated, disease, as the absence of RUBICON conferred a survival advantage in B6.Sle1.*Yaa* mice (Figure 1A). The complete deletion of *Rubicon* is required for this protection (Figure 1A). *Rubicon*^+/–^ B6.Sle1.*Yaa* mice had no differences in mortality compared with WT B6.Sle1.*Yaa* controls (Supplemental Figure 2). Consistent with the prior literature (34), male and female *Cybb*-deficient MRL.Fas*^lpr^* mice had a reduced life span compared with *Cybb*-sufficient controls (34, 35, 37) as 50% or more of the experimental *Cybb^–/–^* cohort did not survive until the experimental endpoint of 16–18 weeks (Figure 1, B and C). Similar to the B6.Sle1.*Yaa* SLE model, no *Rubicon*-deficient MRL.Fas*^lpr^* mice died in the analyzed cohorts (Figure 1, B and C). Strikingly, *Rubicon* deficiency increased survival in male *Cybb^–/y^* mice (Figure 1B). However, this protection was not observed in female *Cybb^–/–^* *Rubicon^–/–^* MRL*.*Fas*^lpr^* mice (Figure 1C).

### Rubicon deficiency reduces SLE renal disease.

**Figure 2:**
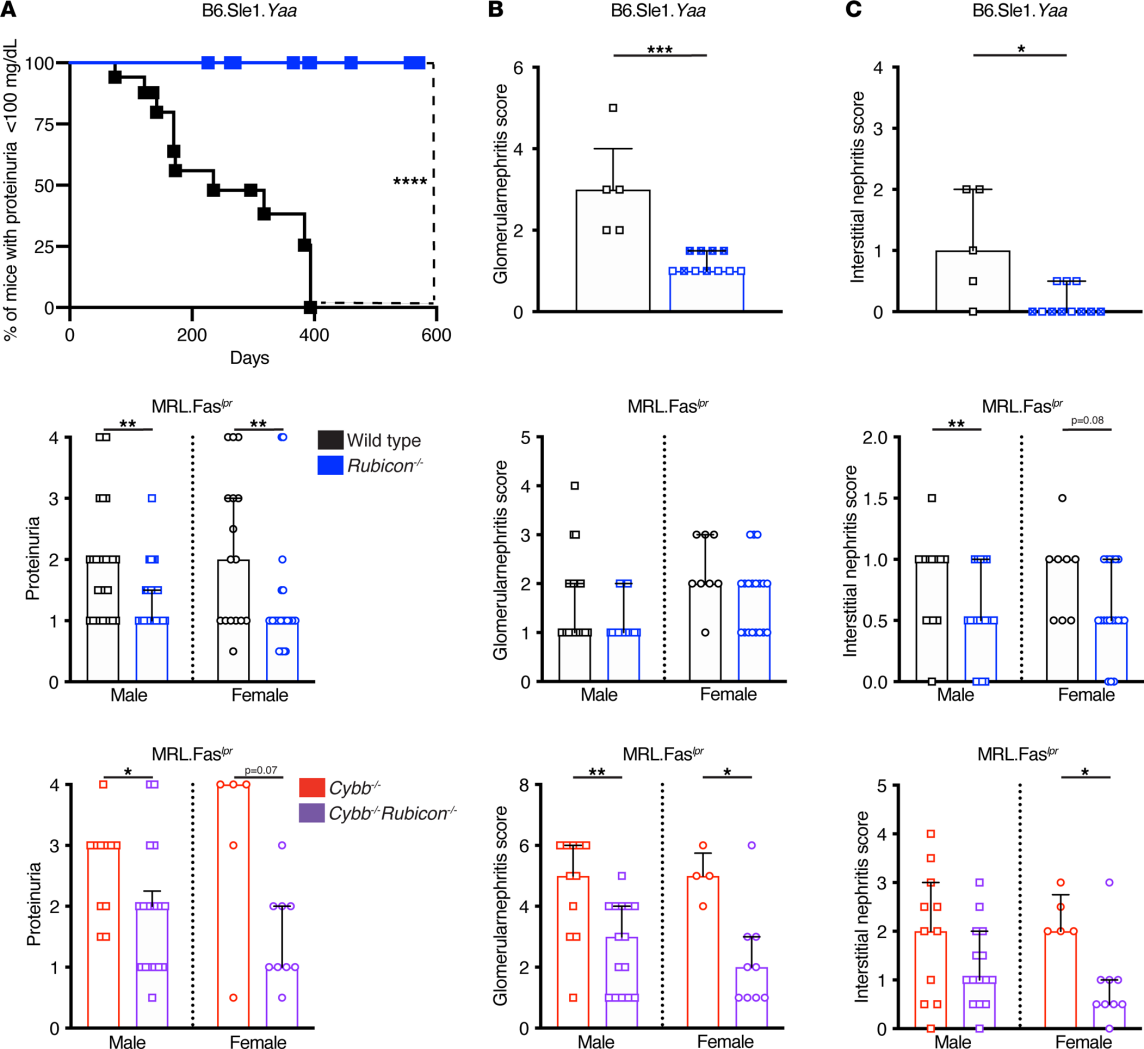
*Rubicon* deficiency protects MRL.Fas*^lpr^* and B6.Sle1.*Yaa* SLE mice from renal disease. (**A**) Kaplan-Meier plot depicting onset of proteinuria in B6.Sle1.*Yaa* mice (top). Proteinuria scores in MRL.Fas*^lpr^* mice (middle and bottom) (B6.Sle1.*Yaa* WT and *Rubicon^+/–^* males *n* = 17; B6.Sle1.*Yaa* *Rubicon^–/–^* males *n* = 28; MRL.Fas*^lpr^* WT males *n* = 27; MRL.Fas*^lpr^* WT females *n* = 16; MRL.Fas*^lpr^ Rubicon^–/–^* males *n* = 29; MRL.Fas*^lpr^ Rubicon^–/–^* females *n* = 27; MRL.Fas*^lpr^ Cybb^–/y^* males *n* = 11; MRL.Fas*^lpr^ Cybb^–/–^* females *n* = 5; MRL.Fas*^lpr^ Rubicon^–/–^Cybb^–/y^* males *n* = 18; and MRL.Fas*^lpr^ Rubicon^–/–^Cybb^–/–^* females *n* = 9 mice per group). (**B**) Glomerulonephritis (GN) scores. (**C**) Interstitial nephritis (IN) scores. Scores are represented as a function of *Rubicon* and *Cybb* genotype. Renal pathology was evaluated in B6.Sle1.*Yaa* WT and B6.Sle1.*Yaa* *Rubicon^–/–^* mice at 8–11 and 19–21 months of age respectively (x denotes mice > 19 months of age). Proteinuria, GN, and IN were evaluated at 16–18 weeks of age in MRL.Fas*^lpr^* unless otherwise indicated. Bars represent the median ± IQR. A log-rank test was used to determine statistical significance between Kaplan-Meier curves. A Mann-Whitney *U* test was performed to determine statistical significance within each sex. (*P < 0.05, **P < 0.01, ***P < 0.001, ****P < 0.0001; and B6.Sle1.*Yaa* WT and *Rubicon^+/–^* males *n* = 5; B6.Sle1.*Yaa* *Rubicon^–/–^* males *n* = 11; MRL.Fas*^lpr^* WT males *n* = 21; MRL.Fas*^lpr^* WT females *n* = 8; MRL.Fas*^lpr^* *Rubicon^–/–^* males *n* = 19; MRL.Fas*^lpr^* *Rubicon^–/–^* females *n* = 20; MRL.Fas*^lpr^* *Cybb^–/y^* males *n* = 11; MRL.Fas*^lpr^* *Cybb^–/–^* females *n* = 5; MRL.Fas*^lpr^* *Rubicon^–/–^Cybb^–/y^* males *n* = 15; and MRL.Fas*^lpr^* *Rubicon^–/–^Cybb^–/–^* females *n* = 9 mice per group unless otherwise indicated).

Both *Rubicon*-deficient B6.Sle1.*Yaa* and MRL.Fas*^lpr^* mice had reduced urine protein compared with WT controls (Figure 2A). *Rubicon-*deficient B6.Sle1.*Yaa* mice were protected from glomerulonephritis (Figure 2B and Supplemental Figure 3); however, such protection was not observed in MRL.Fas*^lpr^* mice (Figure 2B). Further, interstitial nephritis was ameliorated in *Rubicon^–/–^* male MRL.Fas*^lpr^* and B6.Sle1.*Yaa* mice, with a trend toward reduction in MRL.Fas*^lpr^* female mice (*P* = 0.0844) (Figure 2C and Supplemental Figures 3–5). Strikingly, protection from nephritis was observed in an older cohort of *Rubicon*-KO B6.Sle1.*Yaa* mice aged 19–21 months, nearly twice the age of the WT counterparts used in this study (Figure 2, B and C).

Genetic deletion of *Rubicon* in *Cybb*-deficient MRL.Fas*^lpr^* SLE-prone mice reduced proteinuria in male mice (Figure 2A). A similar reduction in female mice was observed, nearly reaching significance (*P* = 0.07) (Figure 2A). Concordant with the proteinuria data, *Rubicon* deficiency reduced glomerulonephritis in *Cybb*-KO mice (Figure 2B and Supplemental Figures 4 and 5). Interstitial nephritis was reduced in female but not male double KO mice (Figure 2C and Supplemental Figures 4 and 5). Rubicon*^–/–^Cybb^–/y^* male and Rubicon*^–/–^Cybb^–/–^* female SLE-prone mice had decreased composite disease scores compared with *Cybb*-KO counterparts (Supplemental Figure 6A). Hence, despite differences not reaching significance in some cases, overall nephritis as assessed by proteinuria, glomerulonephritis, and interstitial nephritis were all reduced by *Rubicon* deficiency, in both murine lupus models and in the context of concomitant *Cybb* deficiency in MRL.Fas*^lpr^* mice.

### Rubicon deficiency reduces splenomegaly and lymphadenopathy.

Spleen weights were decreased in *Rubicon^–/–^* male and female MRL.Fas*^lpr^* mice (Supplemental Figure 6B). Concordant with these data, total spleen cell counts were reduced in *Rubicon*-deficient B6.Sle1.*Yaa* mice compared with WT controls (Supplemental Figure 6C). Axillary lymph node weights were decreased in male mice and trended toward lower weights in female MRL.Fas*^lpr^ Rubicon^–/–^* mice (*P* = 0.07; Supplemental Figure 6D). *Rubicon* deficiency reduced spleen weight in male *Cybb^–/y^* MRL.Fas*^lpr^* mice but did not affect spleen weight in female mice or LN weight in either sex (Supplemental Figure 6, B and D).

### Rubicon regulates the autoantibody response to RNA, ribonuclear proteins, and cardiolipin.

**Figure 3:**
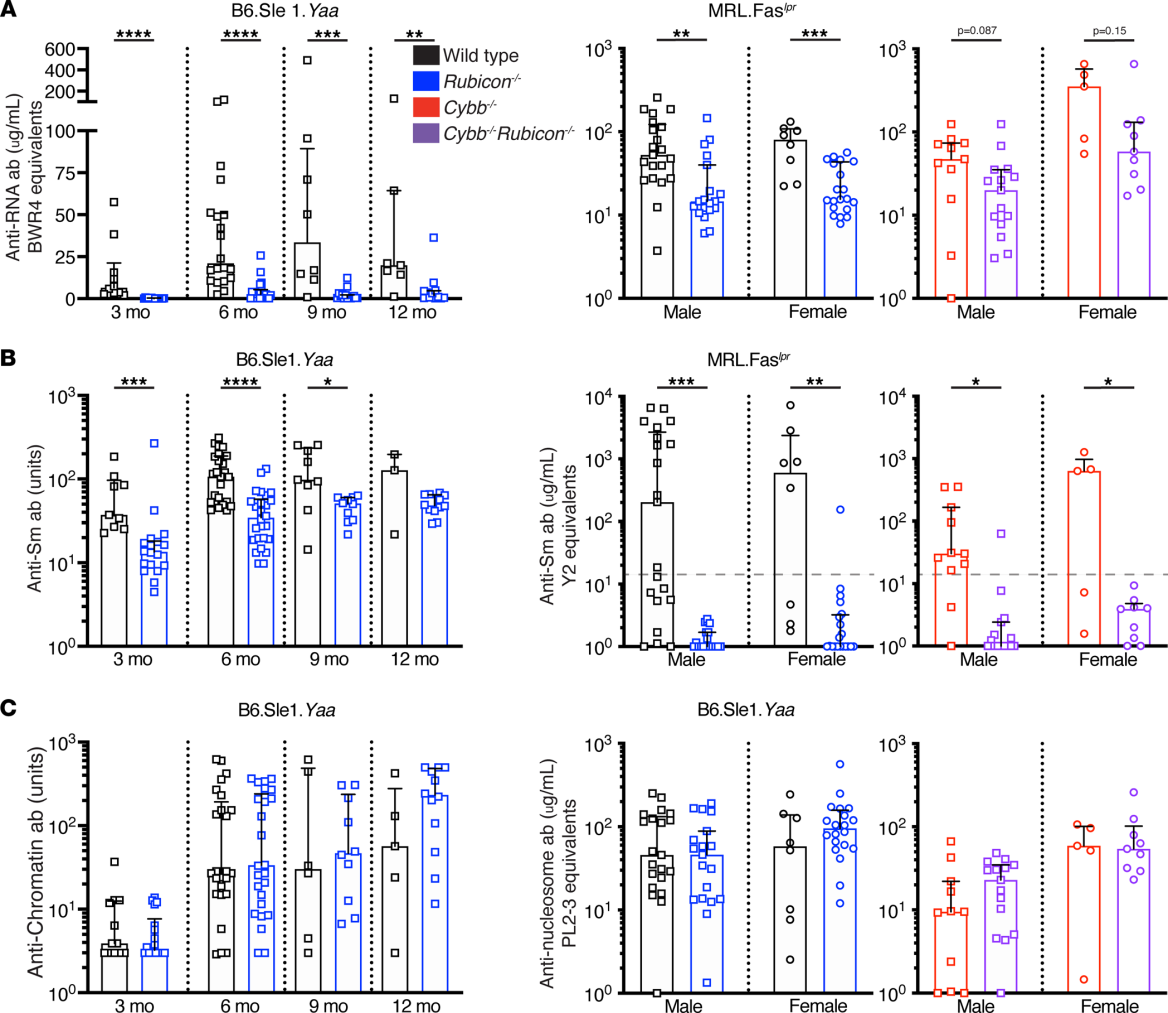
RUBICON regulates the autoantibody response to RNA-associated autoantigens. (**A**) Serum anti-RNA titers in B6.Sle1.*Yaa* (left; 3 months: Control (B6.Sle1.*Yaa* WT and *Rubicon^+/–^*) *n* = 10; B6.Sle1.*Yaa* *Rubicon^–/–^* *n* = 14; 6 months: Control *n* = 20; B6.Sle1.*Yaa* *Rubicon^–/–^* *n* = 20; 9 months: Control *n* = 8; B6.Sle1.*Yaa* *Rubicon^–/–^* *n* = 15; and 12 months: Control *n* = 7; B6.Sle1.*Yaa* *Rubicon^–/–^* *n* = 12) and MRL.Fas*^lpr^* (middle and right ) mice. (**B**) Serum anti-Sm titers in B6.Sle1.*Yaa* (left; 3 months: Control *n* = 9; B6.Sle1.*Yaa* *Rubicon^–/–^* *n* = 18; 6 months: Control *n* = 25; B6.Sle1.*Yaa* *Rubicon^–/–^* *n* = 26; 9 months: Control *n* = 9; B6.Sle1.*Yaa* *Rubicon^–/–^* *n* = 10; and 12 months: Control *n* = 3; B6.Sle1.*Yaa* *Rubicon^–/–^* *n* = 10) and MRL.Fas*^lpr^* (middle and right ) mice. (**C**) Anti-chromatin (B6.Sle1.*Yaa*; 3 months: Control n=12; B6.Sle1.*Yaa* *Rubicon^–/–^* *n* = 18; anti-chromatin titers 6 months: Control *n* = 25; B6.Sle1.*Yaa* *Rubicon^–/–^* *n* = 26; 9 months: Control *n* = 6; B6.Sle1.*Yaa* *Rubicon^–/–^* *n* = 10; and 12 months: Control *n* = 5; B6.Sle1.*Yaa* *Rubicon^–/–^* *n* = 12) or anti-nucleosome (MRL.Fas*^lpr^*) titers in B6.Sle1.*Yaa* (left) and MRL.Fas*^lpr^* (middle and right ) mice. MRL.Fas*^lpr^* antibody titers are represented as a function of *Rubicon* and *Cybb* genotypes at 16–18 weeks of age (MRL.Fas*^lpr^* WT males *n* = 21; MRL.Fas*^lpr^* WT females *n* = 8; MRL.Fas*^lpr^* *Rubicon^–/–^* males *n* = 19; MRL.Fas*^lpr^* *Rubicon^–/–^* females *n* = 20; MRL.Fas*^lpr^* *Cybb^–/y^* males *n* = 11; MRL.Fas*^lpr^* *Cybb^–/–^* females *n* = 5; MRL.Fas*^lpr^* *Rubicon^–/–^Cybb^–/y^* males *n* = 15; and MRL.Fas*^lpr^* *Rubicon^–/–^Cybb^–/–^* females *n* = 9 mice per group). Bars represent the median ± IQR. A Mann-Whitney *U* test was performed to determine statistical significance within each sex unless otherwise indicated. Dashed lines represent the limit of detection of the anti-Sm ELISA. A Fisher’s exact test was performed to determine statistical significance for anti-Sm titers in MRL.Fas*^lpr^* mice (*P < 0.05, **P < 0.01, ***P < 0.001, ****P < 0.0001).

*Rubicon* deficiency reduced anti-RNA titers in both male B6.Sle1.*Yaa* (Figure 3A) and in both male and female MRL.Fas*^lpr^* mice (Figure 3A). A similar trend was observed in male *Rubicon*-deficient, *Cybb*-deficient MRL.Fas*^lpr^* mice (Figure 3A). Differences may have been more significant were it not for the early deaths of the presumably sickest *Cybb*-KO mice (Figure 1, B and C), as serum collection was only performed at the experimental endpoint.

Anti-Smith (anti-Sm) titers were reduced in B6.Sle1.*Yaa* male *Rubicon^–/–^* mice at 3, 6, and 9 months of age (Figure 3B) and in female B6.Sle1 *Rubicon^–/–^* mice at 9 months of age (Supplemental Figure 7A). Strikingly, anti-Sm autoantibodies were absent from male *Rubicon^–/–^* MRL.Fas*^lpr^* mice, and only 1 of 19 female *Rubicon^–/–^* MRL.Fas*^lpr^* mice had a positive anti-Sm titer (Figure 3B). Similarly, *Rubicon* deficiency abolishes the anti-Sm response in male *Cybb-deficient* lupus-prone mice, and only 1 of 7 female *Rubicon^–/–^Cybb^–/–^* female MRL.Fas*^lpr^* mice had positive anti-Sm titers (Figure 3B). Similarly, B6. Sle1.*Yaa* male and B6. Sle1 female *Rubicon^–/–^* mice had reduced anti-cardiolipin antibody titers at 3, 6, and 9 months of age compared with *Rubicon-*sufficient controls (Supplemental Figure 7B). Intriguingly, no differences in anti-chromatin (B6.Sle1.*Yaa*) or anti-nucleosome (MRL.Fas*^lpr^*) titers were identified between any of the groups in MRL.Fas*^lpr^* or B6.Sle1.*Yaa* mice (Figure 3C). By contrast, anti-chromatin titers were markedly attenuated in female B6.Sle *Rubicon-*KO mice (Supplemental Figure 7C).

### Rubicon promotes autoreactive germinal center reactions.

**Figure 4:**
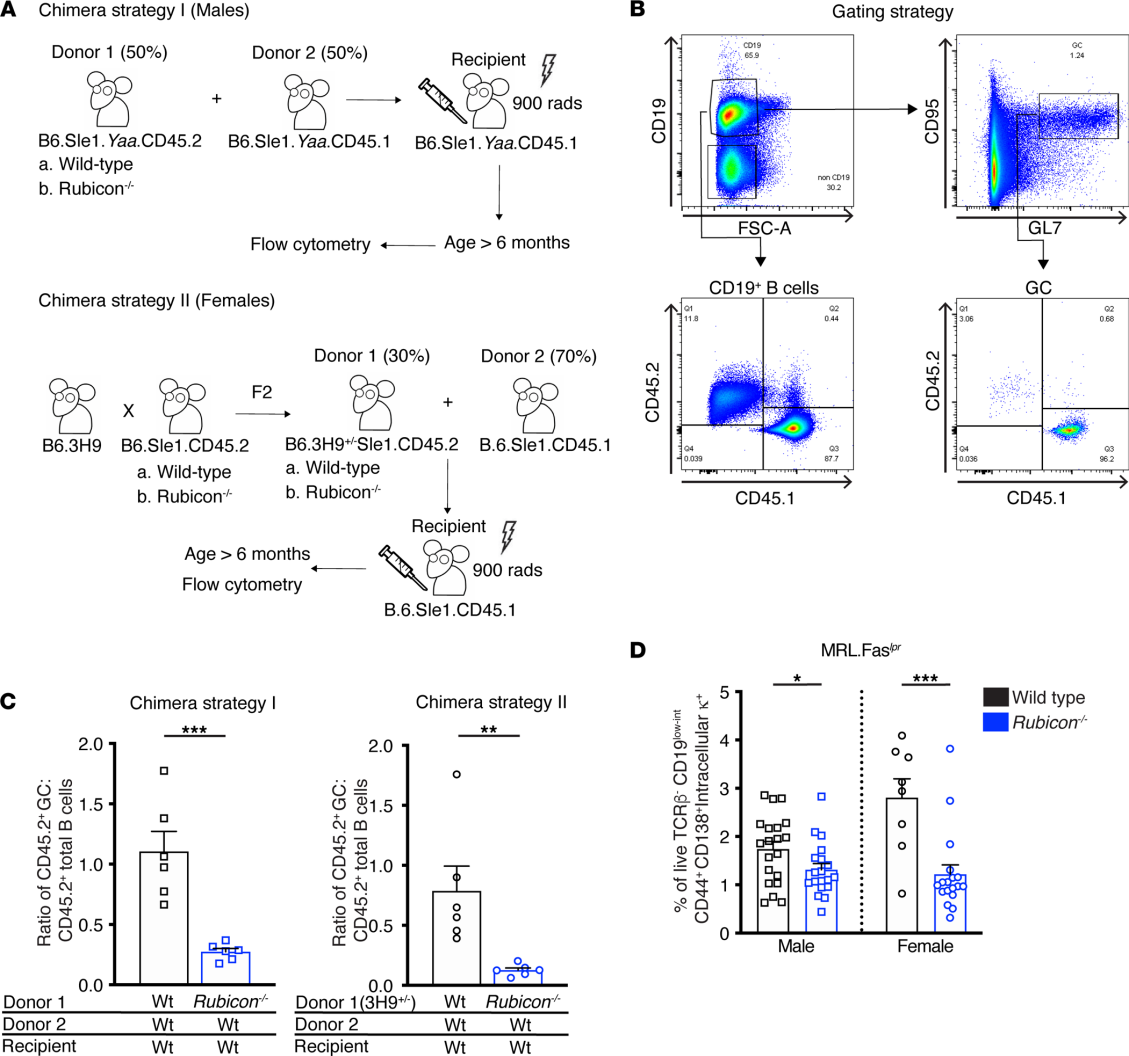
RUBICON is necessary for the germinal center reaction. (**A**) (Top) Mixed bone marrow chimeras were generated with male B6.Sle1.*Yaa* CD45.2 WT or *Rubicon*-KO and CD45.1 B6.Sle1.*Yaa* WT donors. CD45.1 B6.Sle1.*Yaa* irradiated recipients were reconstituted with the aforementioned donors at a 50:50 ratio. Mice were aged for more than 6 months until the presence of anti-chromatin antibodies were detected by ELISA, at which time the mice were euthanized. Reconstitution of the splenic B cell and GC compartments were analyzed by FACS. (Bottom) Mixed bone marrow chimeras were generated by reconstituting female irradiated CD45.1 B6.Sle1 recipients with CD45.2 3H9 B6.Sle1 *Rubicon*-sufficient or -deficient and CD45.1 B6.Sle1 WT donors at a ratio of 1:2. Mice were analyzed as in **A**, top. (**B**) FACS gating strategy for total splenic B cell (CD19^+^) and GCs (CD19^+^CD95^+^GL7^+^). CD45.1 and CD45.2 congenic markers were used to differentiate each donor. (**C**) Ratio of the fraction of CD45.2 GC B cells to the fraction of CD45.2 total B cells in the conventional (strategy I) and 3H9 (strategy II) mixed bone marrow chimeras (*n* = 6 per group). (**D**) Percentages of live cells that are TCRβ^–^ CD44^+^ CD138^+^ intracellular κ^+^ AFCs in spleens of WT or *Rubicon*-KO MRL.Fas*^lpr^* mice (MRL.Fas*^lpr^* WT males *n* = 20; MRL.Fas*^lpr^* WT females *n* = 8; MRL.Fas*^lpr^* *Rubicon^–/–^* males *n* = 19; and MRL.Fas*^lpr^* *Rubicon^–/–^* females *n* = 18 mice per groups). Bars represent the mean ± SEM. A Student’s *t* test was performed to determine statistical significance (*P < 0.05, **P < 0.01, ***P < 0.001).

Germinal center (GC) formation is associated with lupus progression in B6.Sle1.*Yaa* mice, although it is challenging to determine if GCs are autoreactive in this context. To address whether *Rubicon* affects autoreactive GC evolution, we generated conventional bone marrow chimeras using male CD45 congenic B6.Sle1.*Yaa* mice (Figure 4A) in which the dominant autoantibody specificity is anti-RNA. To further examine the anti-chromatin response in female mice, we generated a bone marrow chimera system using the 3H9 transgenic locus (Figure 4A). The 3H9 heavy chain pairs with specific light chains to confer affinity to ssDNA, dsDNA, and cardiolipin but not RNA antigens (54–59). In nonautoimmune mice, tolerance mechanisms prevent the enrichment of these B cells (59–61). However, in autoimmune mouse strains, this is not the case, resulting in a population of autoreactive B cells that can enter GCs (60–63). Here, we crossed 3H9.Sle1 transgenic mice to either *Rubicon*-sufficient or -deficient B6.Sle1 SLE-prone mice, generating donors in which a large fraction of B cells were autoreactive. Mixed bone marrow chimeras were made using the resulting 3H9^+/–^Sle1.CD45.2 *Rubicon-*sufficient or -deficient and B6.Sle1 CD45.1 mice (Figure 4A). At 6 months of age, the B cell compartment in both chimera systems were assessed by FACS (Figure 4B), revealing that *Rubicon*^–/–^ B cells were less able to enter the GC compared with WT controls in both chimera systems (Figure 4C).

Rubicon deficiency had no impact on the total CD4, CD8, or T follicular helper compartments in the conventional chimera system (Supplemental Figure 8, A and B). However, there was a statistically significant reduction in *Rubicon*-KO CD44^+^ CD4 T cells, although this was small in magnitude (Supplemental Figure 8A).

### Rubicon deficiency alters the plasmablast response in SLE mice.

Strikingly, CD19^lo–int^ CD44^+^ CD138^+^ intracellular κ^hi^ antibody forming cells (AFCs) were reduced in *Rubicon*-deficient male and female MRL.Fas*^lpr^* cohorts compared with controls (Figure 4D). There was a trend toward decreased AFCs in double KO male mice (*P* = 0.056) but not in female mice (Supplemental Tables 1 and 2). Failure to reach statistical significance in these cohorts is likely due to premature death of *Cybb* single KO mice, which had worse disease and, thus, would likely have had more plasmablasts. *Rubicon* deficiency did not alter the percentage of CD19^+^ total B cells or CD19^+^CD93^–^CD23^+^CD21/35^–^ follicular zone B cells (Supplemental Tables 1 and 2). *Rubicon*-deficient female mice had an increased percentage of or CD19^+^CD93^–^CD23^lo–int^CD21/35^+^ marginal zone B cells, but a similar difference was not observed in other groups (Supplemental Tables 1 and 2).

### Rubicon effects myeloid expansion in female Cybb-deficient SLE-prone mice.

Others and we have previously reported that global NADPH oxidase deficiency leads to an expansion of the myeloid compartment in both autoimmune and nonautoimmune mouse strains (34, 37, 64). In line with previous data, we observed an increased percentage of splenic macrophages in female *Cybb^–/–^* mice compared with controls (Supplemental Table 2). Interestingly, *Rubicon* deficiency reduced the percentage of macrophages in female *Rubicon^–/–^Cybb^–/–^* lupus mice (Supplemental Table 2). *Rubicon* deficiency did not substantially alter splenic neutrophil or DC percentages (Supplemental Tables 1 and 2).

### Rubicon affects T cell activation in the B6.Sle1.Yaa but not MRL.Fas^lpr^ mice.

While no differences in the T cell compartment, including activated CD4 and CD8 T cells, were observed among the groups in the MRL.Fas*^lpr^* cohort (Supplemental Tables 1 and 2), the percentage of CD44^+^ CD4 T cells was reduced in B6.Sle1.*Yaa* *Rubicon^–/–^* mice (Supplemental Table 3). Intriguingly, the percentages of total T cells and CD8^+^ T cells were elevated in B6.Sle1.*Yaa* male *Rubicon^–/–^* mice (Supplemental Table 3). However, it should be noted that the total spleen count in *Rubicon*-KO B6.Sle1.*Yaa* was substantially reduced in B6.Sle1.*Yaa* *Rubicon^–/–^* mice by approximately 5-fold (median of 6.2 × 10^8^ vs. 1.2 × 10^8^) (Supplemental Figure 6C).

### Myeloid IL-10 deficiency does not affect murine lupus.

LAP is postulated to be immunoprotective via the induction of the antiinflammatory cytokine IL-10. To directly test this in the context of lupus, we generated and examined *IL-10^fl/fl^ LysM Cre^+/–^* and control *IL-10^fl/fl^* mice on the MRL.Fas*^lpr^* background. Such mice would specifically lack IL-10 in the macrophages that conduct LAP.

**Figure 5:**
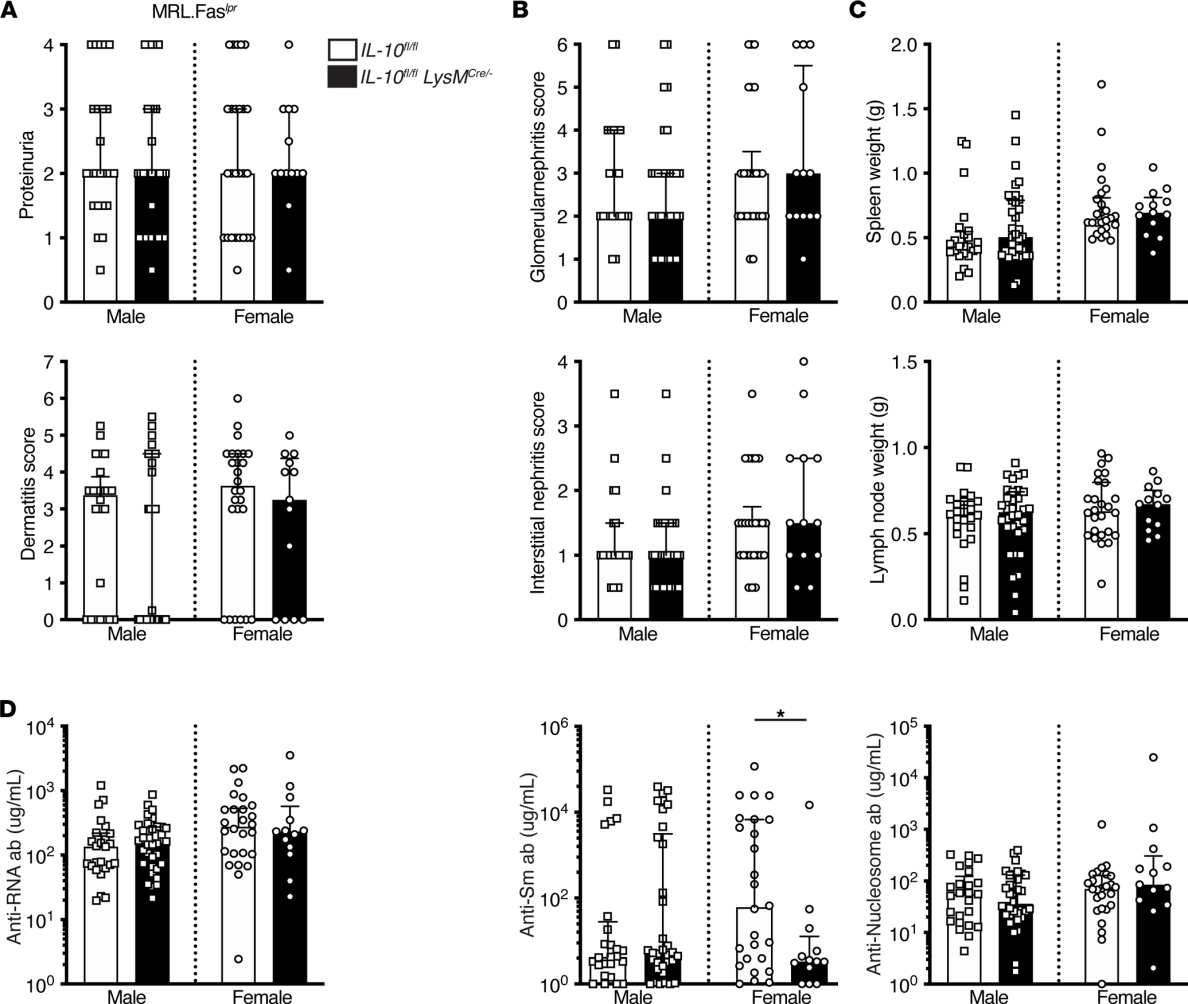
Myeloid IL-10 deficiency does not impact clinical or immunological manifestations of SLE in MRL.Fas*^lpr^* mice. (**A**) Proteinuria (top) and dermatitis scores (bottom). (**B**) Glomerulonephritis (top) and interstitial nephritis (bottom) scores. (**C**) Spleen (top) and axillary lymph node (bottom) weights. (**D**) Anti-RNA (left), anti-SM (middle), and anti-nucleosome (right) antibody titers. Bars represent the median ± IQR. Data are represented as a function of *IL-10^fl/fl^* *LysM Cre* genotype at 16–18 weeks of age (*IL-10^fl/fl^* males *n* = 24 in **A**, *n* = 23 in **B** and **C**, and *n* = 25 in **D**; *IL-10^fl/fl^ LysM^cre/–^* males *n* = 34; *IL-10^fl/fl^* females *n* = 26; and *IL-10^fl/fl^ LysM^cre/–^* females *n* = 13). A Mann-Whitney *U* test was performed to determine statistical significance within each sex unless otherwise indicated. A Fisher’s exact test was performed to determine statistical significance for anti-Sm titers in MRL.Fas*^lpr^* mice (*P < 0.05).

While the hypothesis that LAP induces IL-10 to suppress lupus would predict that *IL-10^fl/fl^ LysM Cre^+/–^* mice would have worse disease, in fact, we did not observe this for any parameter measured. No differences in urine protein, glomerulonephritis, or interstitial nephritis were detected in SLE-prone mice with a myeloid *IL-10* defect (Figure 5, A and B). The incidence of dermatitis was not different across the groups (Figure 5A). No statistically significant differences in splenomegaly or lymphadenopathy were observed (Figure 5C).

IL-10 was efficiently deleted in splenic neutrophils of *IL-10^flf/fl^ LysM-Cre^+/–^* mice (77.67% ± 0.67%) (Supplemental Table 4). Paralleling our prior observation and that of the literature (65), *IL-10* deletion in *IL-10^flf/fl^ LysM-Cre^+/–^* CD11b^+^F4-80^+^ splenic macrophages was 43.48% (± 2.62%) (Supplemental Table 4). As expected, *IL-10* deletion in CD19^+^ B was below the limit of detection (Supplemental Table 4). There was no correlation between neutrophil or macrophage *IL-10* deletion efficiency and either proteinuria score, glomerulonephritis score, or interstitial nephritis score (Supplemental Figure 9).

Myeloid IL-10 genotype did not substantially alter the autoantibody response, with 1 exception (Figure 5D): female *IL-10^fl/fl^LysM Cre^+/–^MRL.*Fas*^lpr^* mice had lower anti-Sm titers than *IL-10*–sufficient controls (Figure 5D). Myeloid IL-10 deficiency did not impact immune composition in the setting of SLE as no differences in splenic B cells, T cells, macrophages, neutrophils, or DC subsets were identified across all groups (Supplemental Table 5).

### Neither Rubicon nor Cybb is required for LAP.

**Figure 6:**
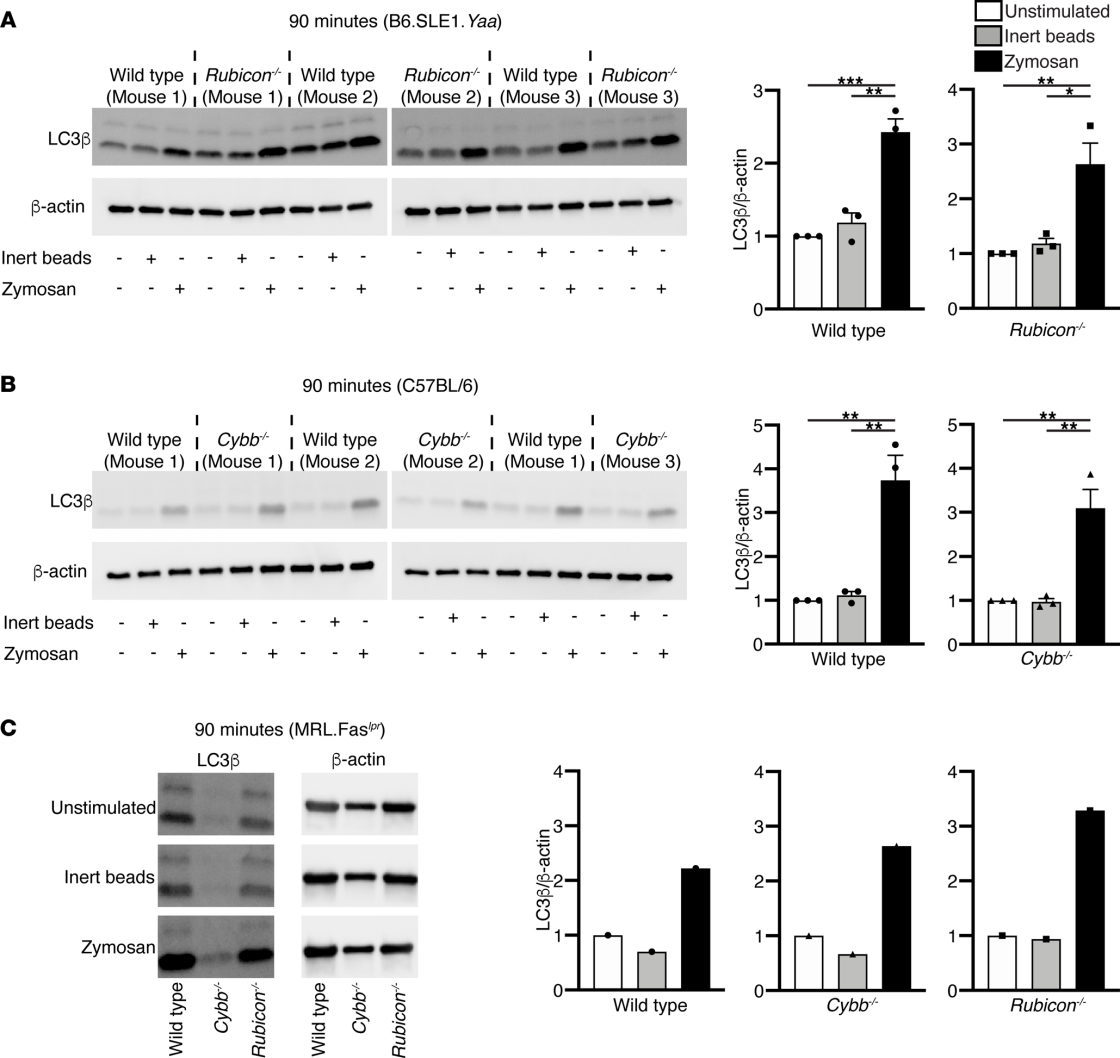
*Rubicon-* and *Cybb*-deficient macrophages can undergo LAP. (**A**) BMDMs generated from *Rubicon*-sufficient and -deficient B6.Sle1.*Yaa* or (**B**) BMDMs generated from *Cybb*-sufficient or -deficient C57BL/6 mice were left untreated, stimulated with inert polystyrene-BSA beads (8:1 beads/cell) or stimulated with zymosan bioparticles (8:1 particles/cell) for 90 minutes. LC3β-I (top band) and LC3β-II (bottom band) were analyzed by IB. LC3β-II bands were quantitated by densitometry and normalized to β-actin loading controls. These ratios were then normalized to the unstimulated condition within each genotype, given a value of 1. Bars represent the mean ± SEM. A 1-way ANOVA with post hoc Tukey’s test was performed to determine statistical significance (*n* = 3 per group). (**C**) BMDMs generated from 6–8 week old WT, *Cybb^–/–^*, and *Rubicon*^–/–^ MRL.Fas*^lpr^* mice were stimulated and analyzed as in **A**. Bars represent normalized densitometry measurements for an individual experiment. (*P < 0.05, **P < 0.01, ***P < 0.001).

The finding that RUBICON and CYBB have opposite effects on lupus-like disease was unexpected, since both proteins are thought to be required for LAP, and the absence of LAP is thought to promote lupus (22, 38). This caused us to revisit the requirement of each of these proteins for LAP itself. We investigated these roles in both nonautoimmune (B6) and autoimmune (B6.Sle1.*Yaa* and MRL.Fas*^lpr^*) genetic backgrounds. To this end, bone marrow–derived macrophages (BMDMs) or peritoneal macrophages were produced from each of the genetic backgrounds and incubated with zymosan-containing particles to stimulate LAP. We chose 60–90 minute time points as LAP is reported to peak in zymosan-stimulated macrophages within this period (40, 41). Cell lysates were then assayed by Western blot for the presence of lipidated LC3β (LC3β-II), a key molecular event in LAP (22, 41). As expected, zymosan-containing particles caused robust LC3β-II accumulation in WT B6 and B6.Sle1.*Yaa* mice (Figure 6, A and B; and Supplemental Figure 10). However, unexpectedly, it also caused similar accumulation in mice deficient in either RUBICON (Figure 6A and Supplemental Figure 10) or CYBB (Figure 6B). Similar findings were obtained with BMDMs derived from MRL.Fas*^lpr^* mice lacking RUBICON or CYBB (Figure 6C and Supplemental Figure 11). Robust LC3β-II accumulation was sustained in RUBICON- and CYBB-deficient BMDMs in both autoimmune and nonautoimmune strains at 180 minutes after stimulation (Supplemental Figure 12). To address whether the SLE microenvironment affected whether RUBICON or CYBB were required to mediate LAP, we isolated peritoneal macrophages from 18-week-old, diseased MRL*.*Fas*^lpr^* mice. Again, LC3β-II was induced in WT and both *Cybb*- and *Rubicon*-KO mice (Supplemental Figure 13). Hence, in contrast to prior reports, we could find no evidence that either CYBB or RUBICON was required for zymosan-induced LC3β-II accumulation in macrophages, a central assay for the detection of LAP.

## Discussion

NADPH oxidase–deficient SLE-prone mice develop more severe SLE than NADPH oxidase–sufficient counterparts and die prematurely (34, 35, 37). Here, we sought to determine whether exacerbated disease in the setting of NADPH oxidase deficiency is due to a failure in LAP, which was reported to require NADPH oxidase (22) and is thought to normally protect from lupus via immunologically silent degradation of dead cells (38). To address this question, we genetically deleted *Rubicon*, another gene that was reported as a required mediator of LAP, in 2 SLE mouse models (22). If the LAP hypothesis were correct, the genetic deletion of *Rubicon* should phenocopy *Cybb* deficiency in SLE-prone mice and exacerbate the lupus phenotype. In addition, the deletion of both genes in the same pathway should have given a similar phenotype to each of the single phenotypes. Our actual findings stood in marked contrast to this model: *Rubicon*-deficient B6.Sle1.*Yaa* and MRL*.*Fas*^lpr^* mice did not develop worsened clinical or immunological manifestations of SLE, as is the case for *Cybb*-deficient MRL*.*Fas*^lpr^* mice. Rather, *Rubicon* deletion increased survival, reduced nephritis, and decreased autoantibody production in B6.Sle1.*Yaa* lupus mice. Moreover, Rubicon deficiency increased survival and ameliorated both glomerulonephritis and interstitial nephritis in *Cybb^–/–^* SLE-prone mice. Thus, we have established RUBICON as a regulatory molecule in SLE pathogenesis.

These unexpected results led us to probe whether RUBICON and CYBB are essential for LAP, as had been reported by Martinez and colleagues (22). To test if *Rubicon^–/–^* and *Cybb^–/–^* MRL.Fas*^lpr^* macrophages were deficient in LAP, we stimulated macrophages with the canonical LAP inducer, zymosan bioparticles. To our surprise, zymosan bioparticles induced the lipidation of LC3β-I to form LC3β-II, a molecular readout for the induction of LAP, in macrophages from both young prediseased and aged *Rubicon^–/–^* and *Cybb^–/–^* MRL.Fas*^lpr^* mice and in macrophages from *Cybb^–/–^* and *Rubicon^–/–^* C57BL/6 mice. Taken together, while we confirm the phenomenon of LAP, our studies highlight a potential flaw with the current view of the proteins required for this process. Importantly, our work indicates that RUBICON may be working through a LAP-independent mechanism to augment SLE pathogenesis.

As our data indicate that CYBB and RUBICON are not required for LAP, the role of LAP in SLE remains ambiguous. Production of IL-10 downstream of dead cell engulfment is thought to be a primary mechanism by which LAP protects against the immune response to the self. In fact, our group identified macrophages and T cells, but not B cells, as major producers of IL-10 in murine lupus (66). To determine the role of myeloid IL-10, and by extension LAP, in the in vivo disease setting, we genetically deleted *IL-10* in neutrophils and macrophages in MRL*.*Fas*^lpr^* mice by a Cre-lox approach, utilizing *LysM-Cre*. *LysM-Cre*–mediated *IL-10* deletion did not alter SLE pathogenesis. Because LysM-Cre is not efficient in all macrophage populations (65), it is possible that partial deletion of *IL-10* in the myeloid compartment is not sufficient to modulate SLE. However, we believe this explanation to be less likely for 2 reasons. First, both *IL10^–/–^* and *IL10^+/–^* MRL*.*Fas*^lpr^* mice developed more severe renal disease and dermatitis compared with their *IL-10*–intact counterparts, indicating that a 50% reduction in global IL-10 gene dose exacerbates SLE (67). Second, Martinez and colleagues (22, 38) used *LysM-Cre* to target multiple LAP genes in macrophages, which was sufficient to demonstrate both reduced LAP and an autoinflammatory phenotype in these animals. Considering these points, we conclude that myeloid IL-10 production, downstream of LAP or other processes, is not a major regulatory mechanism augmenting systemic autoimmunity in our IL-10–sensitive model (67). Moreover, as our prior (66) and current work do not show a role for myeloid and B cell–derived IL-10 in mediating lupus pathogenesis, by elimination, it is likely that T cell–derived IL-10 is the source of IL-10–regulating disease in MRL*.*Fas*^lpr^* mice.

Strikingly, RUBICON deficiency regulates the formation of antibodies toward RNA, ribonucleoproteins, and cardiolipin autoantigens in the context of SLE. We have previously reported that *Cybb*-deficient MRL.Fas*^lpr^* mice develop elevated anti-RNA and anti-Sm titers (34). Further, *Rubicon* deletion abrogates the anti-Sm response in both *Cybb*-sufficient and -deficient SLE-prone mice. These findings may provide a clue as to how RUBICON deficiency constrains autoimmunity. The autoantibody profile in *Rubicon*-deficient SLE-prone mice resembles the autoantibody response observed in *Tlr7^–/–^* MRL*.*Fas*^lpr^* mice, which lack anti-RNA and anti-ribonucleoprotein but make antibodies to DNA and chromatin (68, 69). Similarly to *Rubicon*-deficient SLE-prone mice, *Tlr7*^–/–^ MRL*.*Fas*^lpr^* mice are protected from renal manifestations of SLE (68, 69). It is thus possible that RUBICON and TLR7 promote disease by similar or interrelated mechanisms. TLR7 and RUBICON both localize to the endosome and it is plausible that RUBICON could regulate TLR7 trafficking or signaling in B cells (22, 70–72). Furthermore, RNA must traffic to the endosome to be detected by endosomal TLR receptors, such as TLR7. One mechanism by which RUBICON could modulate SLE pathogenesis is through trafficking of RNA cargo. Indeed, ATG5, an E3 ligase required for both autophagy and LAP, has been implicated in RNA trafficking to TLR-containing endosomes in DCs (73). A similar mechanism could deliver RNA cargo to endosomal TLR7s in B cells, thus affecting the development of TLR7-dependent antibodies in SLE. Supporting this idea, B cell–specific deletion of *Atg5* improves both survival and renal disease in the TLR7 transgenic model (TLR7.1 Tg) of SLE (74). Interplay between RUBICON, RNA trafficking, and TLR7 signaling in lupus is, thus, an intriguing possibility suggested by our data.

B6.Sle1.*Yaa* *Rubicon^–/–^* mice transgenic for the 3H9 Vh region that encodes lupus-related autoantibody specificities show a defect in selection of 3H9 B cells into the GC compartment. These findings suggest that RUBICON enables activation of at least some types of self-reactive B cells. Raso et al. connected the loss of integrin α_v_ in B cells with a loss of RUBICON-dependent noncanonical autophagy in B cells (75); however, these studies did not look at global *Rubicon^–/–^* phenotypes in the setting of autoimmunity and failed to convincingly demonstrate that RUBICON was necessary for LC3β-II formation in B cells (Figure 5; ref. 60).

Our chimera data suggest that RUBICON promotes autoimmunity, at least in part, via a B cell–intrinsic mechanism. However, in B6.Sle1.*Yaa* and MRL*.*Fas*^lpr^* disease cohorts, it is not clear in which cell RUBICON is acting. It is plausible that *Rubicon* expression in other cell types, including the myeloid and T cell compartments, contributes to disease pathogenesis. Indeed, the percent of activated CD4^+^ CD44^+^ T cells is statistically reduced in *Rubicon^–/–^* B6.Sle1.*Yaa* mice, though to a very minor absolute degree. However, this was not the case in the MRL*.*Fas*^lpr^* model. These findings, thus, do not provide strong support for *Rubicon* deficiency driving an intrinsic T cell defect. The cell specific role of RUBICON in SLE still requires further investigation.

Importantly, to our knowledge, our data implicate RUBICON as a novel mediator of systemic autoimmunity, an intriguing finding that may have therapeutic implications for patients with SLE. RUBICON and CYBB instead function antagonistically to each other, with CYBB restraining and RUBICON promoting disease, contrary to the original hypothesis that the 2 molecules work in concert to mediate LAP (22). Finally, and of substantial importance to the field, we find that LC3β-II formation, a lynchpin of LAP, does not depend on either CYBB or RUBICON, which should lead to a reevaluation of our fundamental understanding of this process.

## Methods

### Mice.

Rubicon-KO mice on the C57BL/6 background were a gift from Douglas Green (St. Jude Children’s Research Hospital, Memphis, Tennessee, USA). *Rubicon*^–/–^ mice were crossed to the B6.Sle1.*Yaa* strain (The Jackson Laboratory) and genotyped for Dmit15, -17, and -47. Serum was obtained monthly for measurement of autoantibodies and urine was obtained monthly for measurement of proteinuria by dipstick (Multistix, Thermo Fisher Scientific). Groups of mice were euthanized at 8–11 or 19–21 months of age to assess SLE pathology.

*Rubicon*-deficient MRL.Fas*^lpr^* mice were generated by in vitro fertilization and CRISPR/Cas9 technology as previously described by replacing Asp188 with a premature stop codon (22). To generate mice for experimental cohorts, we intercrossed (a) *Rubicon^–/+^Cybb^y/–^* × *Rubicon^–/+^Cybb^+/–^*, (b) *Rubicon^–/+^Cybb^y/+^* × *Rubicon^–/+^Cybb^+/–^*, and (c) *Rubicon^–/+^* × *Rubicon^–/+^*. This breeding produced littermate controls for each group. SLE pathology was assessed at 16–18 weeks of age.

*IL-10^fl/fl^* C57BL/6 mice (76) were backcrossed to the MRL.Fas*^lpr^* strain for at least 9 generations (66). To generate mice for experimental cohorts, we intercrossed *LysM-Cre^+/–^IL-10^fl/fl^* to *IL-10l^fl/fl^*. This breeding allowed us to use littermate controls for each group. SLE pathology was assessed at 18 weeks of age.

All mice were housed under specific pathogen–free conditions.

### Evaluation of SLE pathology.

MRL.Fas*^lpr^* and B6.Sle1.*Yaa* SLE cohorts were analyzed as previously described (34, 77–80).

### Bone marrow chimeras.

CD45.1 Sle1.*Yaa* male and female mice were irradiated and male recipients were reconstituted with mixed bone marrow from CD45.2 Sle1.*Yaa*. *Rubicon^–/–^* and CD45.1 Sle1.*Yaa* donors in a 1:1 ratio. Female recipients received mixed bone marrow from CD45.1 Sle1 and CD45.2 3H9.Sle1. *Rubicon^–/–^* donors in a 2:1 ratio. Recipients were monitored for more than 6 months and were euthanized once anti-chromatin antibodies appeared in the serum.

### Induction of LAP.

To induce LAP, peritoneal macrophages or BMDMs were stimulated with zymosan bioparticles (Thermo Fisher Scientific) at a ratio of 8:1 (particles/cell) at indicated time points. Inert BSA-conjugated polystyrene beads or unstimulated conditions were used as negative controls.

### IB.

Lysates were analyzed by SDS PAGE. Immunodetection was achieved using the following antibodies: LC3β (Cell Signaling, D11, 1:1000), RUBICON (Cell Signaling, D9F7, 1:1000), β-Actin HRP (Cell Signaling, 8H10D10, 1:10,000), and Anti-Rabbit IgG HRP (Cell Signaling, 1:10,000). Proteins were visualized by an ECL chemiluminescence reagent and imaged by a Protein Simple imager.

### Statistics.

Statistical analysis was performed using Prism 8.0 (GraphPad). A log-rank test was used to determine statistical significance between Kaplan-Meier curves. Linear regression was used to determine correlation between disease parameter and deletion efficiency in indicated cell type. A 2-tailed Mann-Whitney *U* test, 2-tailed Student’s *t* test, 2-tailed Welch’s *t* test, 1-way ANOVA with post hoc Tukey’s test, and a Fisher’s exact test were performed where indicated and appropriate. A *P* value of less than 0.05 was considered statistically significant.

### Study approval.

Animal studies were approved by the University of Pittsburgh and Feinstein Institutes for Medical Research Institutional Animal Care Use Committees.

## Author contributions

RAG, CG, CR, AM, WH, RC, and JT performed experiments and analyzed data. SIB performed histopathological evaluation of the kidneys. RAG, AD, and MJS designed experiments and wrote the manuscript. KMN and AMC provided intellectual support.

## Supplementary Material

Supplemental data

## Version 1 02/22/2022

In-Press Preview

## Version 2 04/08/2022

Electronic publication

## References

[1] Lisnevskaia L (2014). Systemic lupus erythematosus. Lancet.

[2] Mahajan A (2016). Clearance deficiency and cell death pathways: a model for the pathogenesis of SLE. Front Immunol.

[3] Han S (2017). A novel subset of anti-inflammatory CD138 ^+^ macrophages is deficient in mice with experimental lupus. J Immunol.

[4] Herrmann M (1998). Impaired phagocytosis of apoptotic cell material by monocyte-derived macrophages from patients with systemic lupus erythematosus. Arthritis Rheum.

[5] Baumann I (2002). Impaired uptake of apoptotic cells into tingible body macrophages in germinal centers of patients with systemic lupus erythematosus. Arthritis Rheum.

[6] Svensson BO (1975). Serum factors causing impaired macrophage function in systemic lupus erythematosus. Scand J Immunol.

[7] Miyanishi M (2007). Identification of Tim4 as a phosphatidylserine receptor. Nature.

[8] Kobayashi N (2007). TIM-1 and TIM-4 glycoproteins bind phosphatidylserine and mediate uptake of apoptotic cells. Immunity.

[9] Mevorach D (2010). Clearance of dying cells and systemic lupus erythematosus: the role of C1q and the complement system. Apoptosis.

[10] Hu CY (2009). Genetic polymorphism in milk fat globule-EGF factor 8 (MFG-E8) is associated with systemic lupus erythematosus in human. Lupus.

[11] Hanayama R (2004). Autoimmune disease and impaired uptake of apoptotic cells in MFG-E8-deficient mice. Science.

[12] Huang W (2017). Milk fat globule-EGF factor 8 suppresses the aberrant immune response of systemic lupus erythematosus-derived neutrophils and associated tissue damage. Cell Death Differ.

[13] Cohen PL (2002). Delayed apoptotic cell clearance and lupus-like autoimmunity in mice lacking the c-mer membrane tyrosine kinase. J Exp Med.

[14] Bedard K, Krause KH (2007). The NOX family of ROS-generating NADPH oxidases: physiology and pathophysiology. Physiol Rev.

[15] Lam GY (2010). The many roles of NOX2 NADPH oxidase-derived ROS in immunity. Semin Immunopathol.

[16] El-Benna J (2008). Priming of the neutrophil NADPH oxidase activation: role of p47phox phosphorylation and NOX2 mobilization to the plasma membrane. Semin Immunopathol.

[17] Bratton DL, Henson PM (2011). Neutrophil clearance: when the party is over, clean-up begins. Trends Immunol.

[18] Frasch SC (2008). NADPH oxidase-dependent generation of lysophosphatidylserine enhances clearance of activated and dying neutrophils via G2A. J Biol Chem.

[19] Arroyo A (2002). NADPH oxidase-dependent oxidation and externalization of phosphatidylserine during apoptosis in Me2SO-differentiated HL-60 cells. Role in phagocytic clearance. J Biol Chem.

[20] Hampton MB (2002). Oxidant-mediated phosphatidylserine exposure and macrophage uptake of activated neutrophils: possible impairment in chronic granulomatous disease. J Leukoc Biol.

[21] Frasch SC (2011). Signaling via macrophage G2A enhances efferocytosis of dying neutrophils by augmentation of Rac activity. J Biol Chem.

[22] Martinez J (2015). Molecular characterization of LC3-associated phagocytosis reveals distinct roles for Rubicon, NOX2 and autophagy proteins. Nat Cell Biol.

[23] Winkelstein JA (2000). Chronic granulomatous disease. Report on a national registry of 368 patients. Medicine (Baltimore).

[24] Landing BH, Shirkey HS (1957). A syndrome of recurrent infection and infiltration of viscera by pigmented lipid histiocytes. Pediatrics.

[25] Schaller J (1972). Illness resembling lupus erythematosus in mothers of boys with chronic granulomatous disease. Ann Intern Med.

[26] Cale CM (2007). Cutaneous and other lupus-like symptoms in carriers of X-linked chronic granulomatous disease: incidence and autoimmune serology. Clin Exp Immunol.

[27] Olofsson P (2003). Positional identification of Ncf1 as a gene that regulates arthritis severity in rats. Nat Genet.

[28] Hultqvist M (2004). Enhanced autoimmunity, arthritis, and encephalomyelitis in mice with a reduced oxidative burst due to a mutation in the Ncf1 gene. Proc Natl Acad Sci U S A.

[29] Jacob CO (2012). Lupus-associated causal mutation in neutrophil cytosolic factor 2 (NCF2) brings unique insights to the structure and function of NADPH oxidase. Proc Natl Acad Sci U S A.

[30] Kim-Howard X (2014). Allelic heterogeneity in NCF2 associated with systemic lupus erythematosus (SLE) susceptibility across four ethnic populations. Hum Mol Genet.

[31] Zhao J (2017). A missense variant in NCF1 is associated with susceptibility to multiple autoimmune diseases. Nat Genet.

[32] Olsson LM (2017). A single nucleotide polymorphism in the NCF1 gene leading to reduced oxidative burst is associated with systemic lupus erythematosus. Ann Rheum Dis.

[33] Kelkka T (2014). Reactive oxygen species deficiency induces autoimmunity with type 1 interferon signature. Antioxid Redox Signal.

[34] Campbell AM (2012). NADPH oxidase inhibits the pathogenesis of systemic lupus erythematosus. Sci Transl Med.

[35] Jacob CO (2017). Haploinsufficiency of NADPH oxidase subunit NCF2 is sufficient to accelerate full-blown lupus in NZM.2328 mice. Arthritis Rheumatol.

[36] Wing K (2015). Germ-free mice deficient of reactive oxygen species have increased arthritis susceptibility. Eur J Immunol.

[37] Kienhofer D (2017). Experimental lupus is aggravated in mouse strains with impaired induction of neutrophil extracellular traps. JCI Insight.

[38] Martinez J (2016). Noncanonical autophagy inhibits the autoinflammatory, lupus-like response to dying cells. Nature.

[39] Fernandez-Boyanapalli R (2010). Impaired phagocytosis of apoptotic cells by macrophages in chronic granulomatous disease is reversed by IFN-γ in a nitric oxide-dependent manner. J Immunol.

[40] Sanjuan MA (2007). Toll-like receptor signalling in macrophages links the autophagy pathway to phagocytosis. Nature.

[41] Martinez J (2011). Microtubule-associated protein 1 light chain 3 alpha (LC3)-associated phagocytosis is required for the efficient clearance of dead cells. Proc Natl Acad Sci U S A.

[43] Hochberg MC (1997). Updating the American College of Rheumatology revised criteria for the classification of systemic lupus erythematosus. Arthritis Rheum.

[44] Woo J (1995). Combined effects of FK506 (tacrolimus) and cyclophosphamide on atypical B220+ T cells, cytokine gene expression and disease activity in MRL/MpJ-lpr/lpr mice. Clin Exp Immunol.

[45] Van Bruggen MC (1998). Attenuation of murine lupus nephritis by mycophenolate mofetil. J Am Soc Nephrol.

[46] Shiraki M (1984). Long term administration of cyclophosphamide in MRL/1 mice. I. The effects on the development of immunological abnormalities and lupus nephritis. Clin Exp Immunol.

[47] Yamamoto K (1990). Experimental treatment of autoimmune MRL-lpr/lpr mice with immunosuppressive compound FK506. Immunology.

[48] Ahuja A (2007). Depletion of B cells in murine lupus: efficacy and resistance. J Immunol.

[49] Wandstrat AE (2004). Association of extensive polymorphisms in the SLAM/CD2 gene cluster with murine lupus. Immunity.

[50] Mohan C (1998). Genetic dissection of SLE pathogenesis. Sle1 on murine chromosome 1 leads to a selective loss of tolerance to H2A/H2B/DNA subnucleosomes. J Clin Invest.

[51] Morel L (2000). Genetic reconstitution of systemic lupus erythematosus immunopathology with polycongenic murine strains. Proc Natl Acad Sci U S A.

[52] Morel L (1994). Polygenic control of susceptibility to murine systemic lupus erythematosus. Immunity.

[53] Subramanian S (2006). A Tlr7 translocation accelerates systemic autoimmunity in murine lupus. Proc Natl Acad Sci U S A.

[54] Shlomchik MJ (1987). Structure and function of anti-DNA autoantibodies derived from a single autoimmune mouse. Proc Natl Acad Sci U S A.

[55] Radic MZ (1991). Ig H and L chain contributions to autoimmune specificities. J Immunol.

[56] Radic MZ (1993). B lymphocytes may escape tolerance by revising their antigen receptors. J Exp Med.

[57] Ibrahim SM (1995). Light chain contribution to specificity in anti-DNA antibodies. J Immunol.

[58] Moisini I (2012). The Yaa locus and IFN-α fine-tune germinal center B cell selection in murine systemic lupus erythematosus. J Immunol.

[59] Erikson J (1991). Expression of anti-DNA immunoglobulin transgenes in non-autoimmune mice. Nature.

[60] Mandik-Nayak L (1997). Regulation of anti-double-stranded DNA B cells in nonautoimmune mice: localization to the T-B interface of the splenic follicle. J Exp Med.

[61] Paul E (2004). Germinal center checkpoints in B cell tolerance in 3H9 transgenic mice. Int Immunol.

[62] Li Y (2002). Anti-DNA B cells in MRL/lpr mice show altered differentiation and editing pattern. J Exp Med.

[63] Boneparth A (2015). TLR7 influences germinal center selection in murine SLE. PLoS One.

[64] Lee K (2011). Spontaneous and aging-dependent development of arthritis in NADPH oxidase 2 deficiency through altered differentiation of CD11b+ and Th/Treg cells. Proc Natl Acad Sci U S A.

[65] Abram CL (2014). Comparative analysis of the efficiency and specificity of myeloid-Cre deleting strains using ROSA-EYFP reporter mice. J Immunol Methods.

[66] Teichmann LL (2012). B cell-derived IL-10 does not regulate spontaneous systemic autoimmunity in MRL.Faslpr mice. J Immunol.

[67] Yin Z (2002). IL-10 regulates murine lupus. J Immunol.

[68] Nickerson KM (2010). TLR9 regulates TLR7- and MyD88-dependent autoantibody production and disease in a murine model of lupus. J Immunol.

[69] Christensen SR (2006). Toll-like receptor 7 and TLR9 dictate autoantibody specificity and have opposing inflammatory and regulatory roles in a murine model of lupus. Immunity.

[70] Zhong Y (2009). Distinct regulation of autophagic activity by Atg14L and Rubicon associated with Beclin 1-phosphatidylinositol-3-kinase complex. Nat Cell Biol.

[71] Matsunaga K (2009). Two Beclin 1-binding proteins, Atg14L and Rubicon, reciprocally regulate autophagy at different stages. Nat Cell Biol.

[72] Petes C (2017). The Toll for trafficking: Toll-like receptor 7 delivery to the endosome. Front Immunol.

[73] Lee HK (2007). Autophagy-dependent viral recognition by plasmacytoid dendritic cells. Science.

[74] Weindel CG (2015). B cell autophagy mediates TLR7-dependent autoimmunity and inflammation. Autophagy.

[75] Raso F (2018). αv Integrins regulate germinal center B cell responses through noncanonical autophagy. J Clin Invest.

[76] Roers A (2004). T cell-specific inactivation of the interleukin 10 gene in mice results in enhanced T cell responses but normal innate responses to lipopolysaccharide or skin irritation. J Exp Med.

[77] Nickerson KM (2013). Exacerbated autoimmunity in the absence of TLR9 in MRL.Fas(lpr) mice depends on Ifnar1. J Immunol.

[78] Tilstra JS (2020). B cell-intrinsic TLR9 expression is protective in murine lupus. J Clin Invest.

[79] Mohan C (1993). Nucleosome: a major immunogen for pathogenic autoantibody-inducing T cells of lupus. J Exp Med.

[80] Kahn P (2008). Prevention of murine antiphospholipid syndrome by BAFF blockade. Arthritis Rheum.

