# Supplementary material for: Rubicon promotes rather than restricts murine lupus and is not required for LC3-associated phagocytosis
Source: JCI Insight. 2022 Apr 8;7(7):e155537. doi: 10.1172/jci.insight.155537 (PMC9057630; doi:10.1172/jci.insight.155537)
Supplement: Supplemental data [file jciinsight-7-155537-s110.pdf]

Supplemental Materials and Methods

Mice

Rubicon knockout mice on the C57BL/6 background were a kind gift from Dr. Douglas Green (St Jude Children's Research Hospital, Memphis, TN). *Rubicon*$^{-/-}$ mice were crossed to the Sle1.*Yaa* (The Jackson Laboratory) strain. Sle1 was genotyped using 3 primer sets as described by Jackson Laboratories (Bar Harbor, ME) and the *Rubicon* mutant was genotyped using an assay designed by Transnetyx (Cordova, TN) (1). Male and female mice, including heterozygous and wild-type littermates, were monitored every two weeks for proteinuria using Multistix (Siemens). Serum was obtained monthly for measurement of autoantibodies. Mice were euthanized at 8-11 months or 9-21 months of age to assess SLE pathology.

The 3H9 transgene (2) was introduced into the Rubicon$^{-/-}$ Sle1.*Yaa* strain. The CD45.1 allotype variant (The Jackson Laboratory) was separately crossed to the Sle1.Yaa strain.

Rubicon-deficient MRL.Fas^{lpr} mice were generated by *in vitro* fertilization and CRISPR-Cas9 technology as previously described by replacing Asp188 with a premature stop codon by homology directed repair (1). To facilitate screening of founder mice and subsequent genotyping, an EcoR1 restriction site was added downstream of the stop codon (1). MRL.Fas^{lpr} pronuclear-stage zygotes were generated by *in vitro* fertilization. Resulting zygotes were microinjected by the Gene Targeting and Transgenic Core (University of Pittsburgh) with capped Cas9 mRNA, a guide RNA targeting the end of exon 5, and a 183-nucleotide-long single stranded oligo DNA nucleotide (ssODN; Integrated DNA Technologies) repair template. Injected zygotes were cultured overnight

and transferred to pseudopregnant CD1 recipient females (Taconic) to obtain potential founder mice. Mice were genotyped by PCR amplification of the target locus and RFLP analysis. Amplified PCR products were subsequently cloned into a sequencing vector (NEB) and sequenced to verify correct targeting. Potential off-targets with two or fewer mismatches were screened, by amplifying each off-target locus with a specific primer pair and sequencing the PCR product in founder mice as previously described (1). The founders were backcrossed to wild-type MRL.Fas^{lpr} mice (The Jackson Laboratory catalogue # 000485) for 2 generations to breed out undetected off-target gene editing events.

Cybb-deficient (Catalogue# 002365) mice were purchased from the Jackson Laboratory and backcrossed to the MRL.Fas^{lpr} strain for at least 9 generations as previously described (3).

To generate mice for experimental cohorts, we intercrossed: (1) *Rubicon*$^{-/+}$*Cybb*$^{y/-}$ X *Rubicon*$^{-/+}$*Cybb*$^{+/-}$, (2) *Rubicon*$^{-/+}$*Cybb*$^{y/+}$ X *Rubicon*$^{-/+}$*Cybb*$^{+/-}$, and (3) *Rubicon*$^{-/+}$ X *Rubicon*$^{-/+}$. This breeding produced littermate controls for each group. SLE pathology was assessed at 16-18 weeks of age.

IL-10$^{fl/fl}$ C57BL/6 mice (4) were backcrossed to the MRL.Fas^{lpr} strain for at least 9 generations (5). Homozygosity for the *lpr* mutation was verified by PCR. *LysM-Cre* MRL.Fas^{lpr} mice were intercrossed with *IL-10*$^{fl/wt}$ MRL.Fas^{lpr} mice. Resulting *LysM-Cre*$^{+/-}$ *IL-10*$^{fl/wt}$ MRL.Fas^{lpr} mice were then crossed with MRL.Fas^{lpr} *IL-10*$^{fl/wt}$. To generate mice for experimental cohorts, we intercrossed *LysM-Cre*$^{+/-}$ *IL-10*$^{fl/fl}$ to *IL-10l*$^{fl/fl}$ mice. This breeding allowed us to use littermate controls for each group. SLE pathology was assessed at 16-18 weeks of age.

Quantitative PCR to assess IL-10 deletion efficiency

To determine the efficiency and specificity of *IL-10* deletion, cell lysates were generated from *LysM-Cre*$^{+/-}$ *IL-10*$^{fl/fl}$ and control FACS sorted splenocytes (in-house lysis buffer: 50mM Tris-Base, 50mM KCl, 0.63mM EDTA, 0.22% Tween-20, 0.22% NP-40, and 1/40 proteinase K). Genomic DNA was used as a qRT-PCR template. qRT-PCR reactions were completed in triplicate using the Kappa SYBR Green QPCR kit and analyzed on a Roche Light Cycler instrument. The amount of *IL-10* in each sample was normalized to the unaffected gene, *Tlr9*. Genomic DNA of the same cell type from *IL-10*$^{fl/fl}$ mice was used as undeleted control.

Evaluation of SLE pathology

MRL.Fas^{lpr} and B6.Sle1.*yaa* cohorts were analyzed as previously described (3, 6). Skin disease was scored based on the extent of lesions on the dorsum of the neck and back. Macroscopic surface area was scored from 0 to 5 for an affected area up to 9.1 cm^2. One additional point was given for the presence of ear (1/4 point each) and muzzle (1/2 point) dermatitis (7). Proteinuria was screened using Albustix (Siemens) or Multistix (Fisher) at indicated time points. Plasma was obtained by cardiac puncture. Kidneys were removed, bisected, formalin-fixed, paraffin embedded, and H&E stained. Kidneys were scored for glomerulonephritis by a clinical pathologist blinded to genotype. A score of 1-6 was assigned as follows: (1) normal kidney; (2) mesangial expansion, mesangial hypercellularity, and patent capillary loops; (3) enlarged glomeruli with moderate endocapillary hypercellularity; (4) as in 3 but with the addition of marked endocapillary hypercellularity and loss of patency of most capillary loops; (5) few glomeruli with necrosis [karyorrhexis] or few active [cellular or fibrocellular] or organized [fibrous] crescents; (6) many active [cellular or

fibrocellular] or organized [fibrous] crescents, necrosis [karyorrhexis], and/or obliteration of glomerular architecture with segmental /global sclerosis) (8). Interstitial nephritis was scored on a scale of 1-4 in a blinded manner by a clinical pathologist. A score of 1-4 was assigned as follows: (1) minimal inflammation [lymphocytes and plasma cells] confined to the perivascular area; (2) expansion of inflammation throughout the interstitial space but maintained in a discrete area; (3) diffuse infiltrates in over 40% of high-powered fields; (4) diffuse infiltrate throughout the entire interstitial space) (8). Representative H&E images of kidneys from the MRL.Fas^{lpr} cohort were acquired using an Olympus IX83 microscope with an Olympus UC90 camera. For the B6.Sle1.*Yaa* cohort , H&E stained kidney sections were digitized by Histowiz and representative images were acquired with their slide viewer software.

To evaluate overall disease severity, we employed a composite disease score. The composite disease score integrates the severity of kidney disease and mortality in the experimental cohort. Scores were defined as follows:

Mile disease (score=1): GN score 1-2 and/or IN score $\leq$ 1.

Moderate disease (score=2): GN score 2.5-3.5 and/or IN score 1.5-2.

Severe disease (score=3): GN a score $\geq$4 and/or IN score $\geq$ 2.5 and/or death prior to the experimental endpoint.

Autoantibody ELISAs

Anti-Sm, anti-nucleosome, and anti-RNA autoantibody ELISAs were performed on serum procured from MRL.Fas^{lpr} cohorts as previously described (3, 9-12). Specific antibodies were

detected with alkaline phosphatase-conjugated goat anti-mouse IgG (Southern Biotech [1030-04]). The monoclonal antibodies Y2, BWR4, or PL2-3 (in-house) were used as standards for the anti-Sm, anti-RNA, and anti-nucleosome measurements respectively.

In B6.Sle1.*Yaa* cohorts, serial sera from mice aged 3, 6, 9, 12 and 16 months were evaluated for antibodies to Sm/RNP, chromatin, and cardiolipin/β2GP1 as previously described (13, 14). Anti-RNA ELISAs were performed as described above.

Bone marrow chimeras

CD45.1 B6.Sle1.*Yaa* male and CD45.1 B6.Sle1 female mice were totally irradiated with 900 rads. 24 hours after irradiation, male recipients received mixed bone marrow from CD45.1 Sle1.*Yaa* and CD45.2 Sle1.*Yaa Rubicon*-sufficient or -deficient donors in a 1:1 ratio. Female recipients received mixed bone marrow from CD45.1 B6.Sle1 and CD45.2 3H9.B6.Sle1 *Rubicon*-sufficient or -deficient donors in a 2:1 ratio. Recipients were serially monitored >6 months for the presence of anti-chromatin antibodies and subsequently euthanized once these antibodies were detected.

Flow cytometry

Flow cytometry was performed as previously described (3). In brief, spleens were homogenized and red blood cells (RBC) were lysed using Ammonium-Chloride-Potassium (ACK) buffer (prepared in-house). Cells were resuspended in Phosphate Buffered Saline (PBS) with 3% calf serum and the FcR-blocking antibody 2.4G2 (in-house). Live/dead discrimination was performed using fixable viability stain 510 (BD), Ghost Dye Violet 510 (Tonbo), or LIVE/DEAD Aqua (Thermofisher). Surface and intracellular staining antibodies are listed in supplemental tables 6

and 7. Cells were fixed in 1% paraformaldehyde or Cytofix/Cytoperm (BD) where appropriate. Data were procured using a LSRII or Fortessa (BD) with FACS DIVA software and analyzed using FlowJo.

Generation of bone marrow derived macrophages (BMDMs)

Femurs, tibias, and/or iliac crests were harvested from MRL.Fas^{lpr} or B6.Sle1.*Yaa* mice of indicated genotypes and bone marrow was isolated by mortar and pestle. Cells were incubated in ACK buffer (Gibco) for RBC lysis. $10X10^6$ bone marrow cells were plated on 100mm petri dishes in 10mL of complete DMEM (Gibco) media supplemented with 10% FCS, 100 units/mL penicillin (Gibco), 100ug/mL streptomycin (Gibco), 2mM L-glutamine (Gibco), 50uM 2-ME (Sigma), 10mM HEPES (Gibco), and 20-30% L929 (ATCC) macrophage colony stimulating factor (MCSF) conditioned media. Media was replaced on day three and adherent macrophages were harvested on day seven. BMDMs were replated and rested for 24 hours prior to experimental use.

Induction of LAP with zymosan bioparticles

LAP was induced using the canonical LAP stimulus zymosan bioparticles, as previously described (1, 15, 16). In brief, peritoneal macrophages or BMDMs were stimulated with zymosan bioparticles (Thermofisher) at a ratio of 8:1 (particles/cell). Unstimulated conditions and/or 3uM-6uM BSA-conjugated polystyrene beads (Spherotech) were used as negative controls.

Immunoblotting

Cells were lysed in 2X llamelli's buffer (Bio-Rad) supplemented with 5% 2-ME and heated to 95°C for 5 minutes. Lysates were analyzed by SDS-PAGE. The following antibodies were used

to detect proteins of interest: LC3β (Cell Signaling; D11, 1:1000), RUBICON (Cell Signaling; D9F7,1:1000), β-Actin HRP (Cell Signaling; 8H10D10, 1:10,000), Anti-Rabbit IgG HRP (Cell Signaling, 1:10,000). Proteins were visualized by ECL chemiluminescence reagent and imaged by a Protein Simple imager.

Supplemental References

1. Martinez J, Malireddi RK, Lu Q, Cunha LD, Pelletier S, Gingras S, et al. Molecular characterization of LC3-associated phagocytosis reveals distinct roles for Rubicon, NOX2 and autophagy proteins. *Nature cell biology.* 2015;17(7):893-906.

2. Erikson J, Radic MZ, Camper SA, Hardy RR, and Weigert MG. Expression of anti-DNA immunoglobulin transgenes in non-autoimmune mice. *Nature.* 1991;349:331-34.

3. Campbell AM, Kashgarian M, and Shlomchik MJ. NADPH oxidase inhibits the pathogenesis of systemic lupus erythematosus. *Science translational medicine.* 2012;4(157):157ra41.

4. Roers A, Siewe L, Strittmatter E, Deckert M, Schluter D, Stenzel W, et al. T cell-specific inactivation of the interleukin 10 gene in mice results in enhanced T cell responses but normal innate responses to lipopolysaccharide or skin irritation. *The Journal of experimental medicine.* 2004;200(10):1289-97.

5. Teichmann LL, Kashgarian M, Weaver CT, Roers A, Muller W, and Shlomchik MJ. B Cell-Derived IL-10 Does Not Regulate Spontaneous Systemic Autoimmunity in MRL.Faslpr Mice. *The Journal of Immunology.* 2012;188(2):678-85.

6. Nickerson KM, Cullen JL, Kashgarian M, and Shlomchik MJ. Exacerbated autoimmunity in the absence of TLR9 in MRL.Fas(lpr) mice depends on Ifnar1. *Journal of immunology.* 2013;190(8):3889-94.

7. Berland R, Fernandez L, Kari E, Han JH, Lomakin I, Akira S, et al. Toll-like receptor 7-dependent loss of B cell tolerance in pathogenic autoantibody knockin mice. *Immunity.* 2006;25(3):429-40.

8. Tilstra JS, John S, Gordon RA, Leibler C, Kashgarian M, Bastacky S, et al. B cell-intrinsic TLR9 expression is protective in murine lupus. *J Clin Invest.* 2020;130(6):3172-87.

9. Nickerson KM, Christensen SR, Shupe J, Kashgarian M, Kim D, Elkon K, et al. TLR9 regulates TLR7- and MyD88-dependent autoantibody production and disease in a murine model of lupus. *Journal of immunology.* 2010;184(4):1840-8.

10. Christensen SR, Kashgarian M, Alexopoulou L, Flavell RA, Akira S, and Shlomchik MJ. Toll-like receptor 9 controls anti-DNA autoantibody production in murine lupus. *The Journal of experimental medicine.* 2005;202(2):321-31.

11. Monestier M, and Novick KE. Specificities and genetic characteristics of nucleosome-reactive antibodies from autoimmune mice. *Molecular immunology.* 1996;33(1):89-99.

12. Blanco F, Kalsi J, and Isenberg DA. Analysis of antibodies to RNA in patients with systemic lupus erythematosus and other autoimmune rheumatic diseases. *Clinical and experimental immunology.* 1991;86(1):66-70.

13. Mohan C, Adams S, Stanik V, and Datta SK. Nucleosome: a major immunogen for pathogenic autoantibody-inducing T cells of lupus. *The Journal of experimental medicine.* 1993;177(5):1367-81.

14. Kahn P, Ramanujam M, Bethunaickan R, Huang W, Tao H, Madaio MP, et al. Prevention of murine antiphospholipid syndrome by BAFF blockade. *Arthritis and rheumatism.* 2008;58(9):2824-34.

15. Sanjuan MA, Dillon CP, Tait SW, Moshiach S, Dorsey F, Connell S, et al. Toll-like receptor signalling in macrophages links the autophagy pathway to phagocytosis. *Nature.* 2007;450(7173):1253-7.

16. Martinez J, Almendinger J, Oberst A, Ness R, Dillon CP, Fitzgerald P, et al. Microtubule-associated protein 1 light chain 3 alpha (LC3)-associated phagocytosis is required for the efficient clearance of dead cells. *Proceedings of the National Academy of Sciences of the United States of America.* 2011;108(42):17396-401.

Supplemental Figures

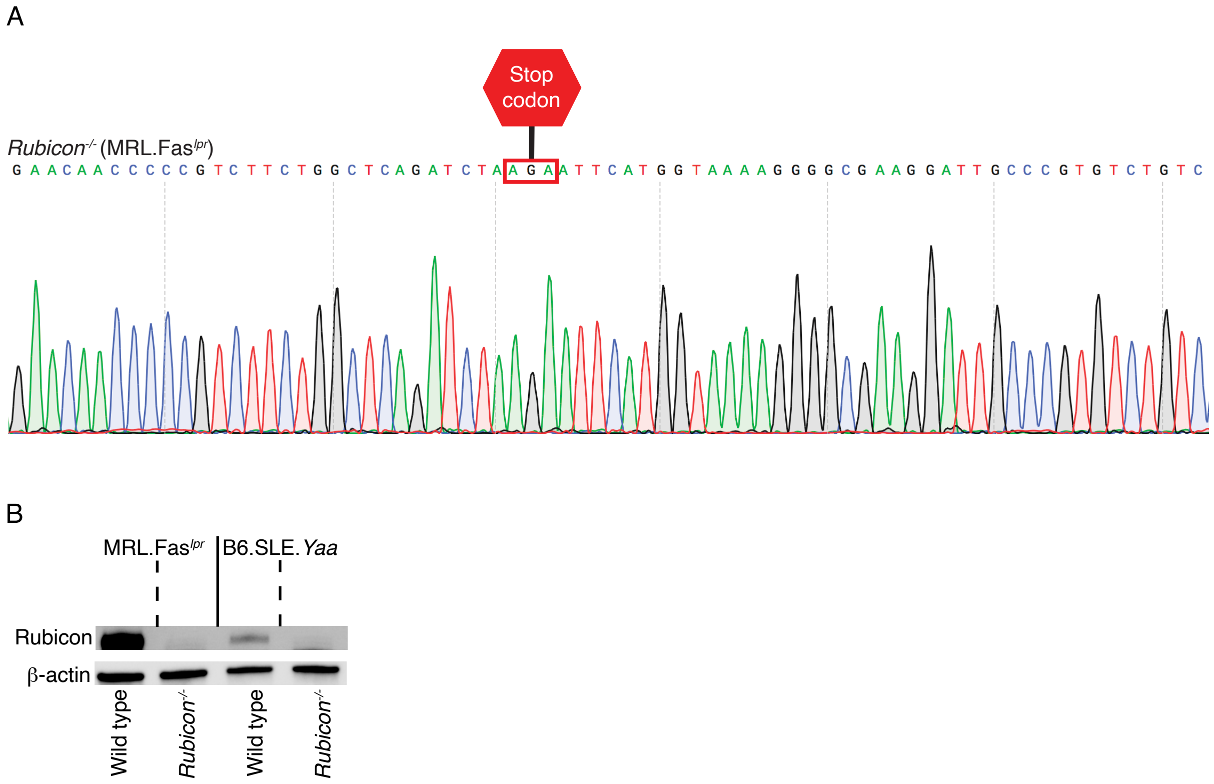

Supplemental Figure 1. Generation of the *Rubicon*-knockout mouse on the MRL.Fas*lpr* background. (A) Sequence of the *Rubicon*-knockout allele generated on the MRL.Fas*lpr* background using *in-vitro* fertilization and CRISRP-Cas9. A premature stop codon was introduced in exon 5 at Asp 188 by using a single-stranded oligonucleotide to facilitate homology directed repair. **(B)** Lysates generated from MRL.Fas*lpr* and B6.Sle1.*Yaa* wild-type or *Rubicon*$^{-/-}$ bone marrow derived macrophages (BMDMs) were solubilized in SDS-PAGE and blotted with indicated antibodies.

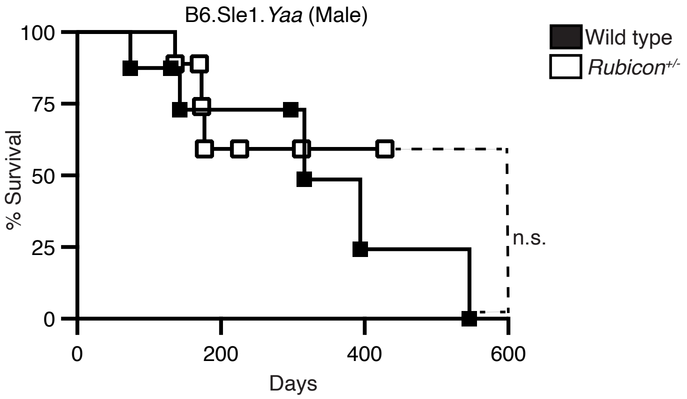

Supplemental Figure 2. *Rubicon* heterozygosity does not confer a survival advantage in SLE prone mice. Kaplan Meier survival curves for B6.Sle1.*Yaa* SLE mice of indicated genotypes. A log-rank test was used to determine statistical significance between Kaplan Meier curves (*p<0.05, ** p<0.01, *** p<0.001, ****p<0.0001 and B6.Sle1.*Yaa* wild-type males n=8 and B6.Sle1.*Yaa Rubicon*$^{-/+}$ males n=9).

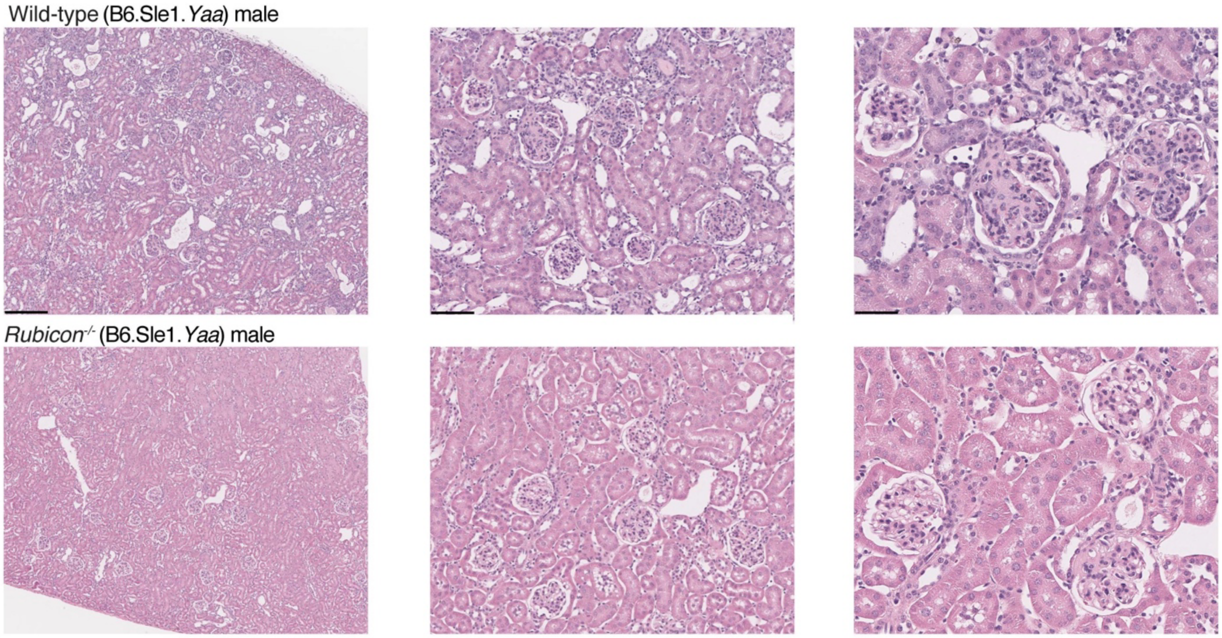

Supplemental Figure 3. *Rubicon* deficiency reduces renal disease in B6.Sle1.*Yaa* SLE mice. Representative images of H&E kidney sections from B6.Sle1.*Yaa* mice of indicated genotypes. Left panel: Original magnification 4X (scale bar: 500uM). Middle panel: 10X (scale bar: 200uM); Left panel: Original magnification 20X (scale bar: 100uM).

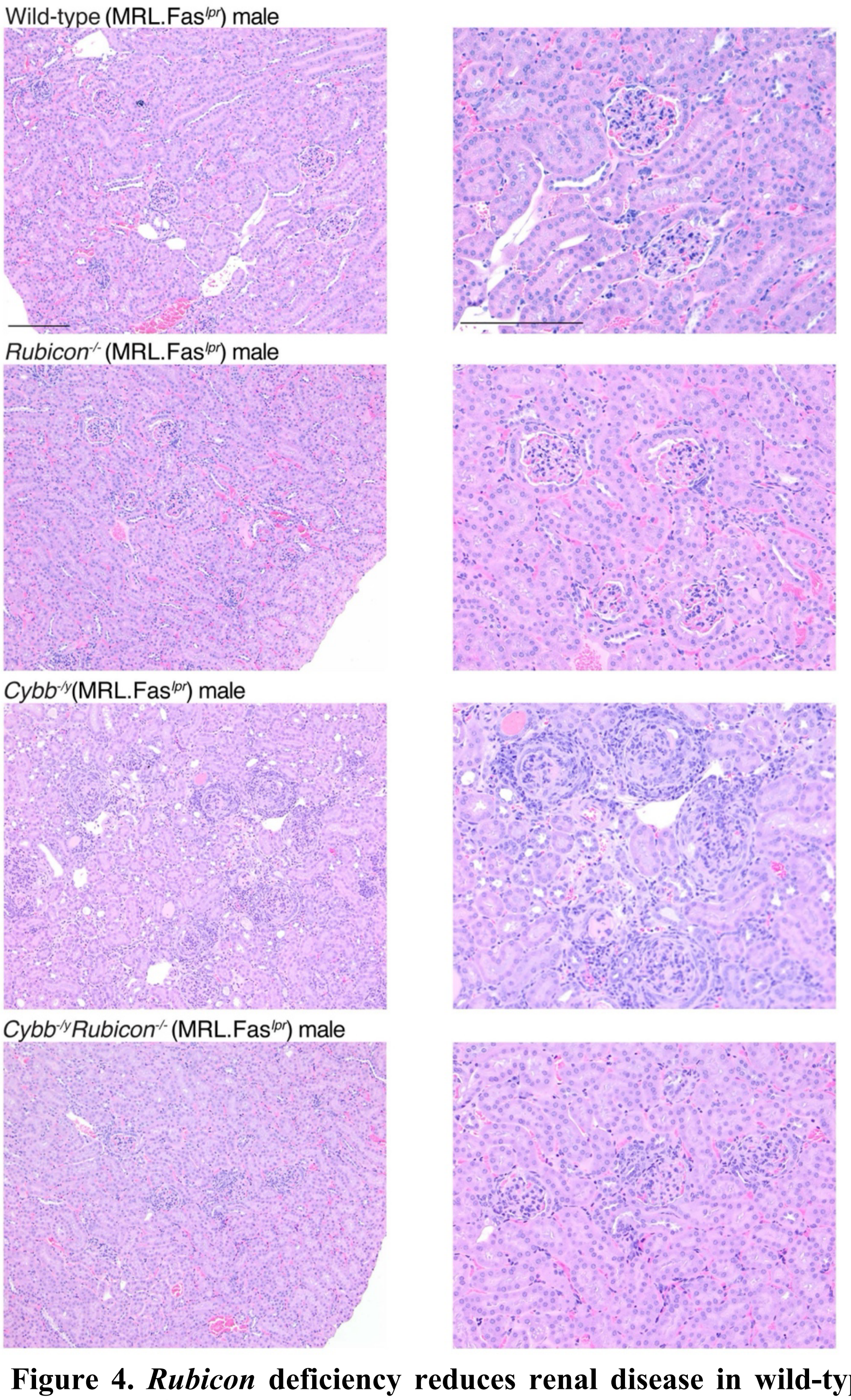

Supplemental Figure 4. *Rubicon* deficiency reduces renal disease in wild-type and *Cybb*-deficient male MRL.Fas^{lpr} mice. Representative images of H&E kidney sections from MRL.Fas^{lpr} mice of indicated genotypes. Left panel: Original magnification 10X. Right panel: Original magnification 20X (scale bars: 200uM).

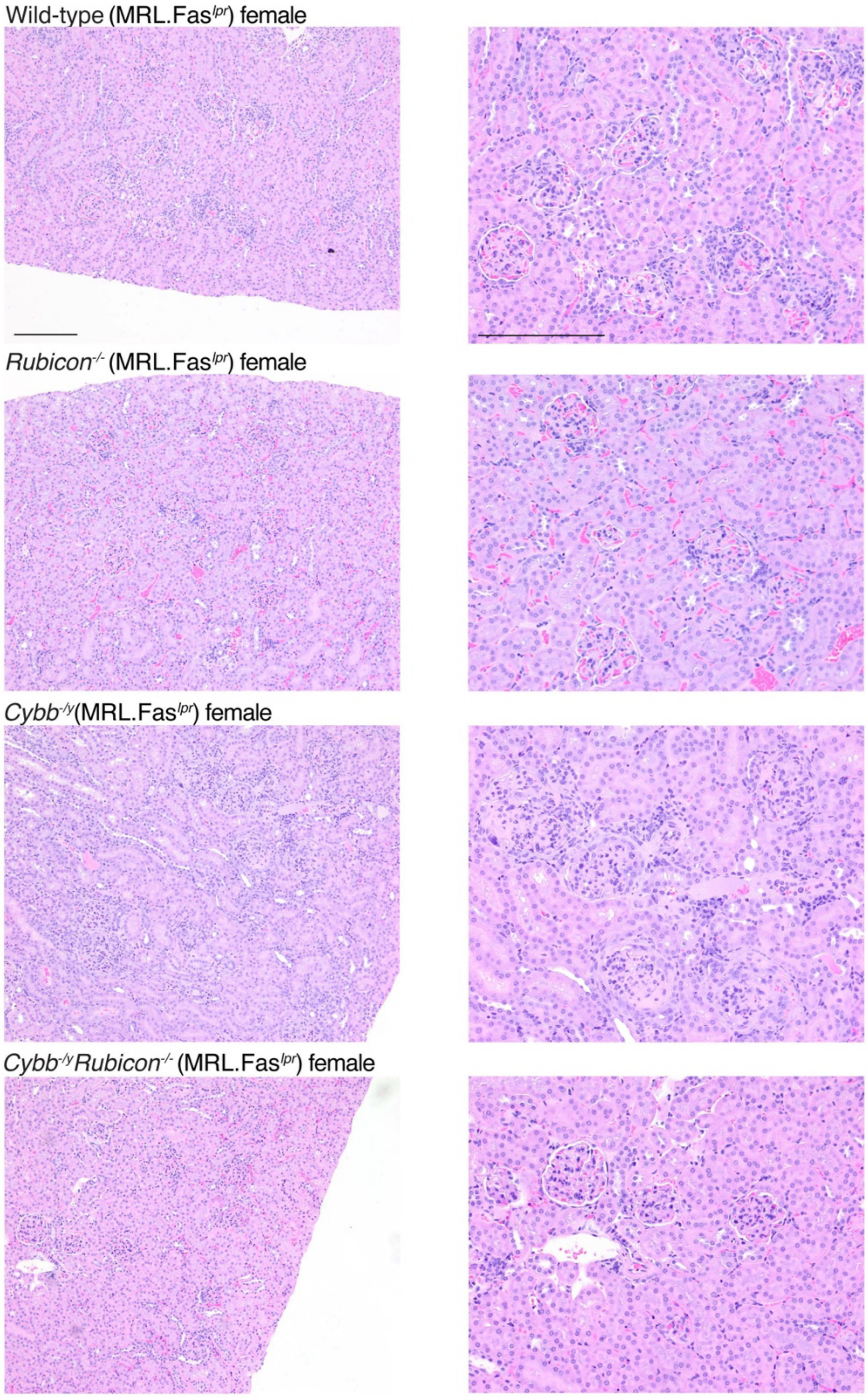

Supplemental Figure 5. *Rubicon* deficiency reduces renal disease in wild-type and *Cybb*-deficient female MRL.Fas^{lpr} mice. Representative images of H&E kidney sections from MRL.Fas^{lpr} mice of indicated genotypes. Left panel: Original magnification 10X. Right panel: Original magnification 20X (scale bars: 200uM).

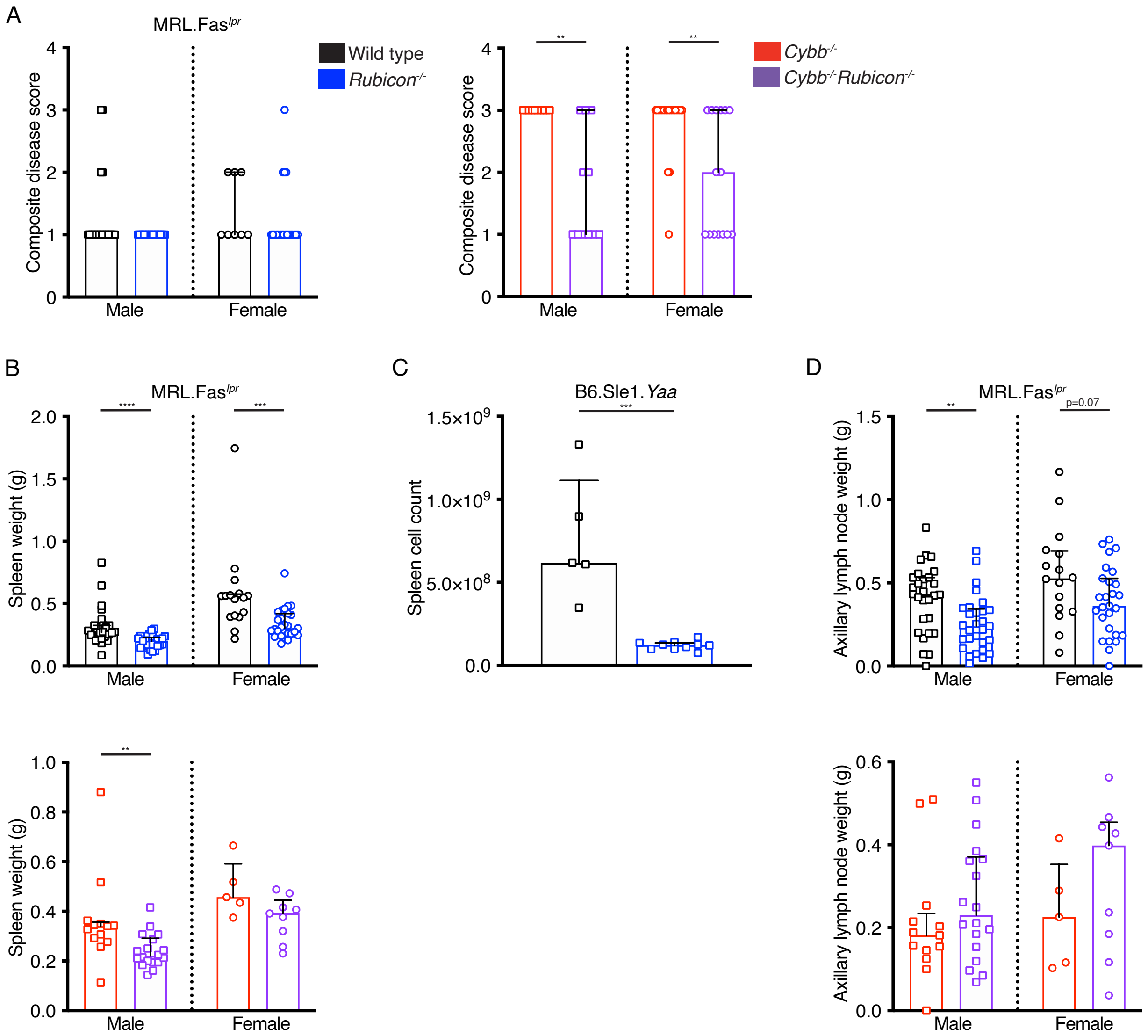

Supplemental Figure 6. *Rubicon* deficiency reduces composite disease score, splenomegaly, and lymphadenopathy. (A) Composite disease score in MRL.Fas^{lpr} mice (MRL.Fas^{lpr} wild-type males n=22; MRL.Fas^{lpr} wild-type females n=8; MRL.Fas^{lpr} *Rubicon*$^{-/-}$ males n=19; MRL.Fas^{lpr} *Rubicon*$^{-/-}$ females n=20; MRL.Fas^{lpr} *Cybb*$^{-/y}$ males n=20; MRL.Fas^{lpr} *Cybb*$^{-/-}$ females n=10; MRL.Fas^{lpr} *Rubicon*$^{-/-}$*Cybb*$^{-/y}$ males n=15; MRL.Fas^{lpr} *Rubicon*$^{-/-}$*Cybb*$^{-/-}$ females n=11 mice per group). **(B)** Spleen weights in MRL.Fas^{lpr} mice. **(C)** Spleen cell counts in B6.Sle1.*Yaa* wild-type (n=5) and B6.Sle1.*Yaa Rubicon*$^{-/-}$ (n=10) mice at 8-11 and 19-21 months of age respectively). **(D)** Lymph node weights in MRL.Fas^{lpr} mice. Disease parameters are represented as a function of *Rubicon* and *Cybb* genotype at 16-18 weeks of age unless otherwise indicated (MRL.Fas^{lpr} wild-type males n=28; MRL.Fas^{lpr} wild-type females n=16; MRL.Fas^{lpr} *Rubicon*$^{-/-}$ males n=29; MRL.Fas^{lpr} *Rubicon*$^{-/-}$ females n=27; MRL.Fas^{lpr} *Cybb*$^{-/y}$ males n=13; MRL.Fas^{lpr} *Cybb*$^{-/-}$ females n=5; MRL.Fas^{lpr} *Rubicon*$^{-/-}$*Cybb*$^{-/y}$ males n=18; MRL.Fas^{lpr} *Rubicon*$^{-/-}$*Cybb*$^{-/-}$ females n=9 mice per group). Bars represent the median ± IQR. A Mann-Whitney U test was performed to determine statistical significance within each gender (*$p<0.05$, ** $p<0.01$, *** $p<0.001$, ****$p<0.0001$).

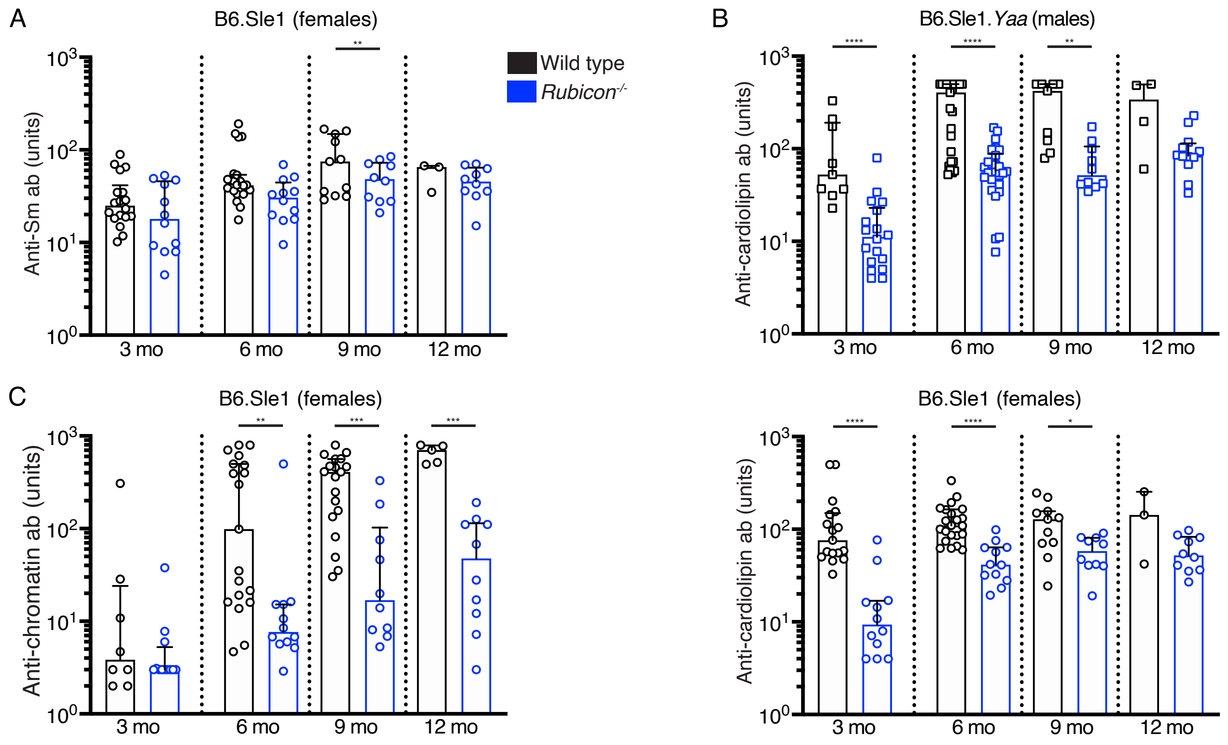

Supplemental Figure 7. RUBICON regulates the autoantibody response to anti-cardiolipin in B6.Sle1.*Yaa* male and B6.Sle1 female mice. (A) Serum anti-Sm titers (3 months: Control (B6.Sle1 wild-type and *Rubicon*$^{+/-}$) n=18; B6.Sle1 *Rubicon*$^{-/-}$ n=12; 6 months: Control n=22; B6.Sle1 *Rubicon*$^{-/-}$ n=12; 9 months: Control n=11; B6.Sle1 *Rubicon*$^{-/-}$ n=10; 12 months: Control n=3; B6.Sle1 *Rubicon*$^{-/-}$ n=10). **(B)** Serum anti-cardiolipin titers in B6.Sle1.*Yaa* (top panel; 3 months: Control (B6.Sle1.*Yaa* wild-type and *Rubicon*$^{+/-}$) n=9; B6.Sle1.*Yaa Rubicon*$^{-/-}$ n=18; 6 months: Control n=25; B6.Sle1.*Yaa Rubicon*$^{-/-}$ n=26; 9 months: Control n=9; B6.Sle1.*Yaa Rubicon*$^{-/-}$ n=10; 12 months: Control n=4; B6.Sle1.*Yaa Rubicon*$^{-/-}$ n=12) and B6.Sle1 (bottom panel; 3 months: Control n=17; B6.Sle1 *Rubicon*$^{-/-}$ n=12; 6 months: Control n=22; B6.Sle1 *Rubicon*$^{-/-}$ n=12; 9 months: Control n=11; B6.Sle1 *Rubicon*$^{-/-}$ n=10; 12 months: Control n=3; B6.Sle1 *Rubicon*$^{-/-}$ n=10) mice**. (C)** Serum anti-chromatin titers (3 months: Control n=8; B6.Sle1 *Rubicon*$^{-/-}$ n=12; 6 months: Control n=19; B6.Sle1 *Rubicon*$^{-/-}$ n=12; 9 months: Control n=19; B6.Sle1 *Rubicon*$^{-/-}$ n=10; 12 months: Control n=5; B6.Sle1 *Rubicon*$^{-/-}$ n=10). Titers are represented as a function of *Rubicon* genotype at indicated time points in B6.Sle1.*Yaa* male or B6.Sle1 female mice. Bars represent the median ± IQR. A Mann-Whitney U test was performed to determine statistical significance within each gender (*p<0.05, ** p<0.01, *** p<0.001, ****p<0.0001).

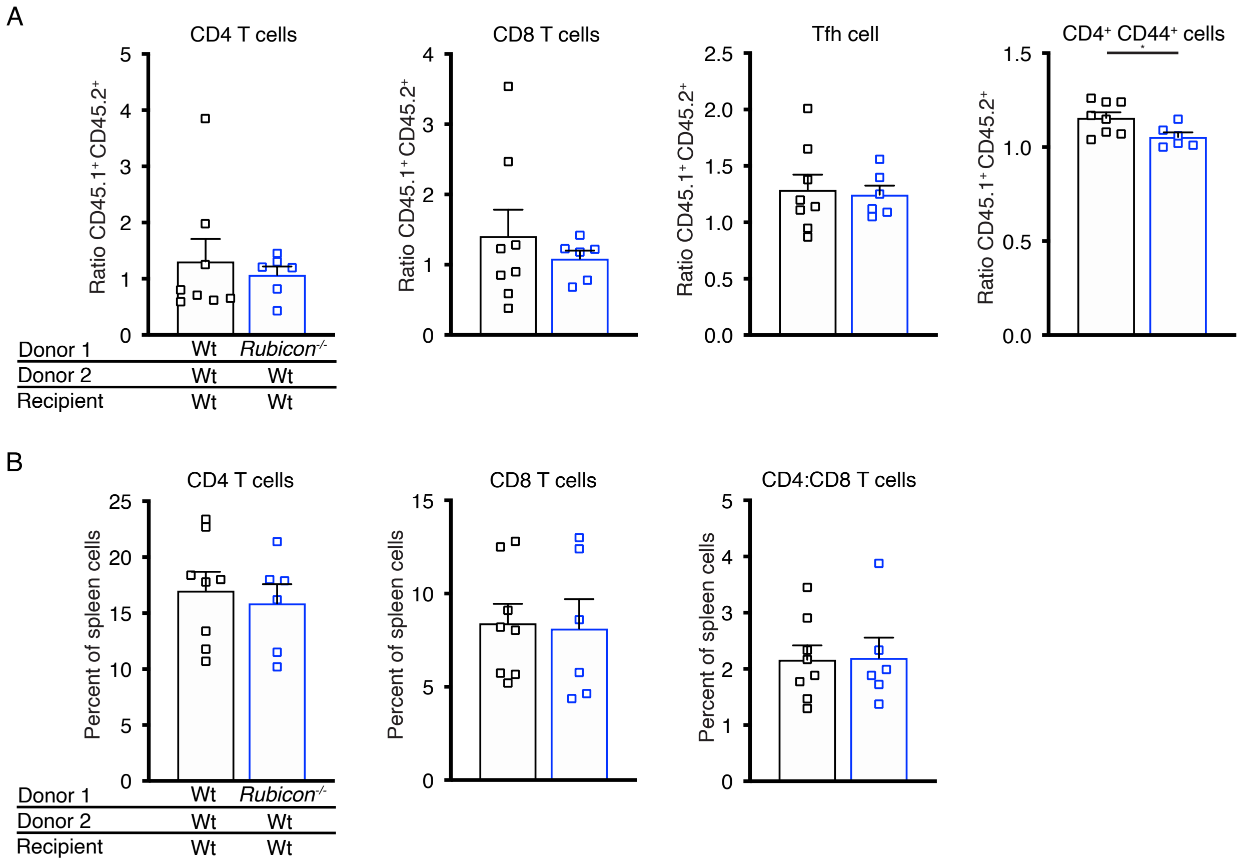

Supplemental Figure 8. RUBICON has a minor effect on T cell activation in B6.Sle1.*Yaa* SLE mice. Mixed bone marrow chimeras were generated with male B6.Sle1.*Yaa* CD45.2 wild-type or *Rubicon*-knockout and CD45.1 B6.Sle1.*Yaa* wild-type donors. CD45.1 B6.Sle1.*Yaa* irradiated recipients were reconstituted with the aforementioned donors at a 50/50 ratio. Mice were aged for > 6 months until the presence of anti-chromatin antibodies were detected by ELISA at which time the mice were euthanized. Reconstitution of splenic T cells was analyzed by FACS. **(A)** Ratio of CD45.1 to CD45.2 splenic $CD4^+$, $CD8^+$, Tfh, and $CD44^+$ CD4 T cells in mixed bone marrow chimeras. **(B)** Percentages of live cells that are $CD4^+$, $CD8^+$, or $CD4^+$: $CD8^+$ in spleens from the mixed bone marrow chimeras (wild type donor n=8 per group; *Rubicon*$^{-/-}$ donor n=6 per group). Bars represent the mean ± SEM. A Student T test was performed to determine statistical significance (*p<0.05, ** p<0.01, *** p<0.001, ****p<0.0001).

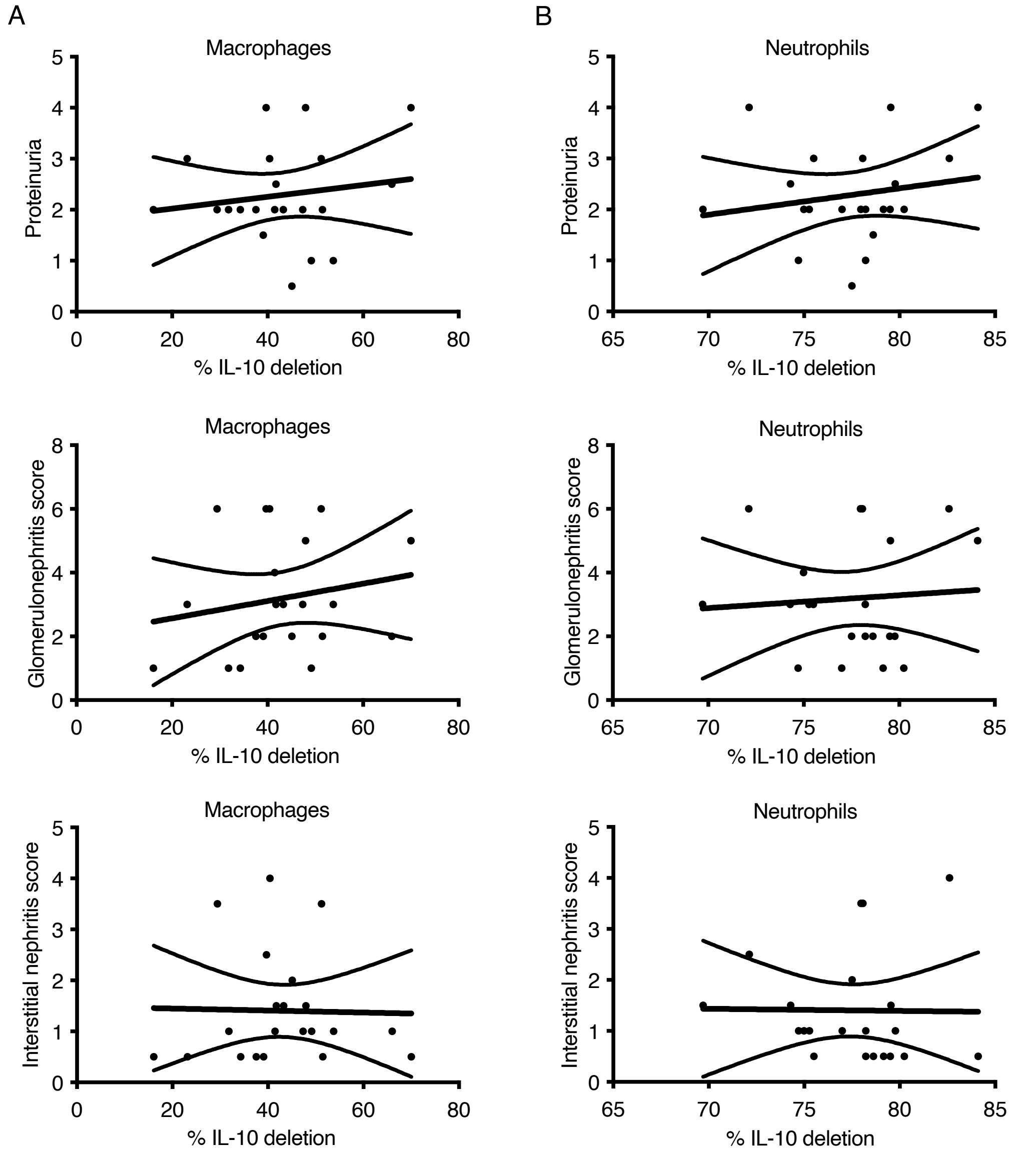

Supplemental Figure 9. Myeloid *IL-10* deletion efficiency does not correlate with SLE renal disease. (A) Correlation between proteinuria (top panel), glomerulonephritis (middle panel), or interstitial nephritis (bottom panel) and percent IL-10 deficiency in MRL.Fas^{lpr} *IL-10*$^{fl/fl}$ *LysM*$^{cre/-}$ macrophages (n=21). **(B)** Correlation between proteinuria (top panel), glomerulonephritis (middle panel), and interstitial nephritis (bottom panel) and percent IL-10 deficiency in MRL.Fas^{lpr} *IL-10*$^{fl/fl}$ *LysM*$^{cre/-}$ neutrophils (n=21). Linear regression was used to determine correlation between disease parameter and deletion efficiency in indicated cell type. Doted curves represent the 95% confidence interval.

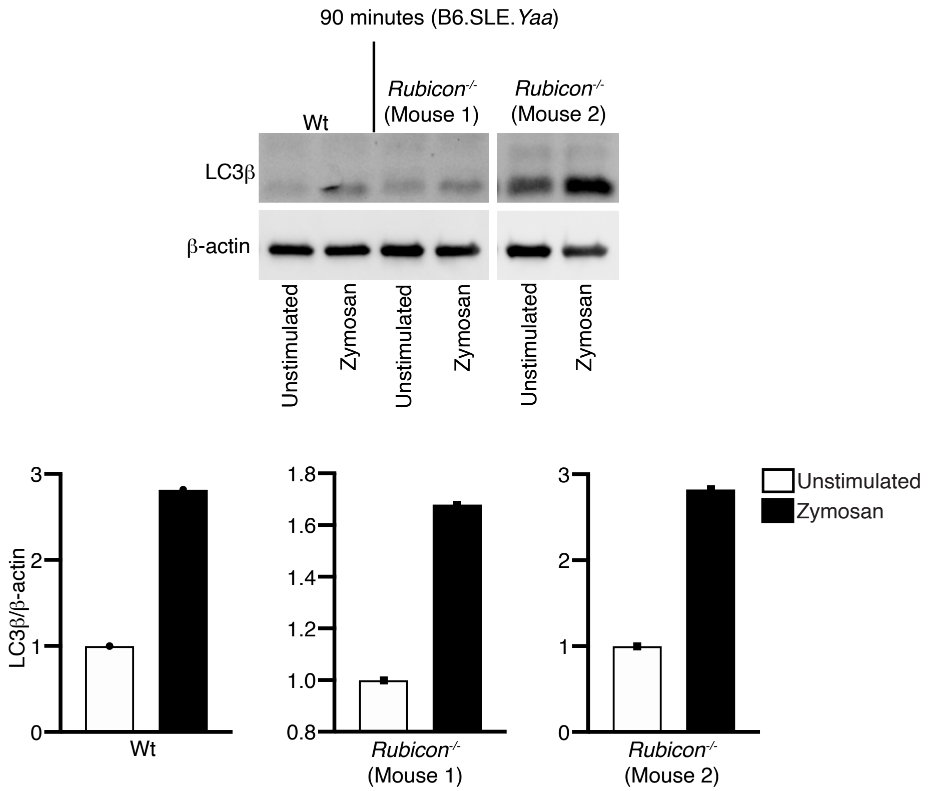

Supplemental Figure 10. *Rubicon* knockout bone marrow derived macrophages from B6.Sle1.*Yaa* mice can undergo LC3-assocated phagocytosis at 90 minutes post-stimulation. Bone marrow derived macrophages (BMDMs) generated from (A) *Rubicon*- sufficient (n=1) and -deficient (n=2) B6.Sle1.*Yaa* mice were left untreated or stimulated with zymosan bioparticles (8 particles: 1 cell) for 90 minutes. LC3β–I (top band) and LC3β–II (bottom band) were analyzed by immunoblot. LC3β–II bands were quantitated as described in Figure 6.

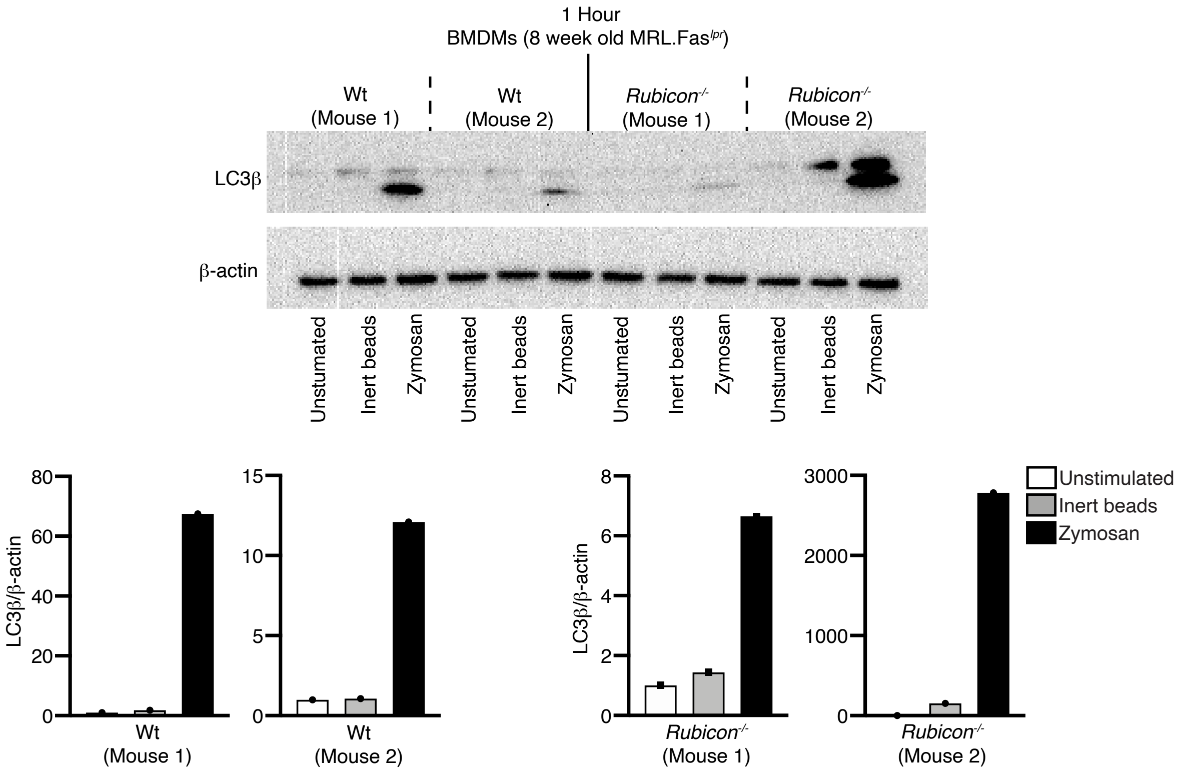

Supplemental Figure 11. *Rubicon$^{-/-}$* bone marrow derived macrophages from 6-8 week old MRL.Fas^{lpr} mice can undergo LC3-assocated phagocytosis at 60 minutes post-stimulation. Bone marrow derived macrophages (BMDMs) generated from *Rubicon*- sufficient (n=2) and -deficient (n=2) 6-8 week old MRL.Fas^{lpr} mice were left untreated, stimulated with inert polystyrene-BSA beads (8 beads: 1 cell), or stimulated with zymosan bioparticles (8 particles: 1 cell) for 60 minutes. LC3β–I (top band) and LC3β–II (bottom band) were analyzed by immunoblot. LC3β–II bands were quantitated as described in Figure 6.

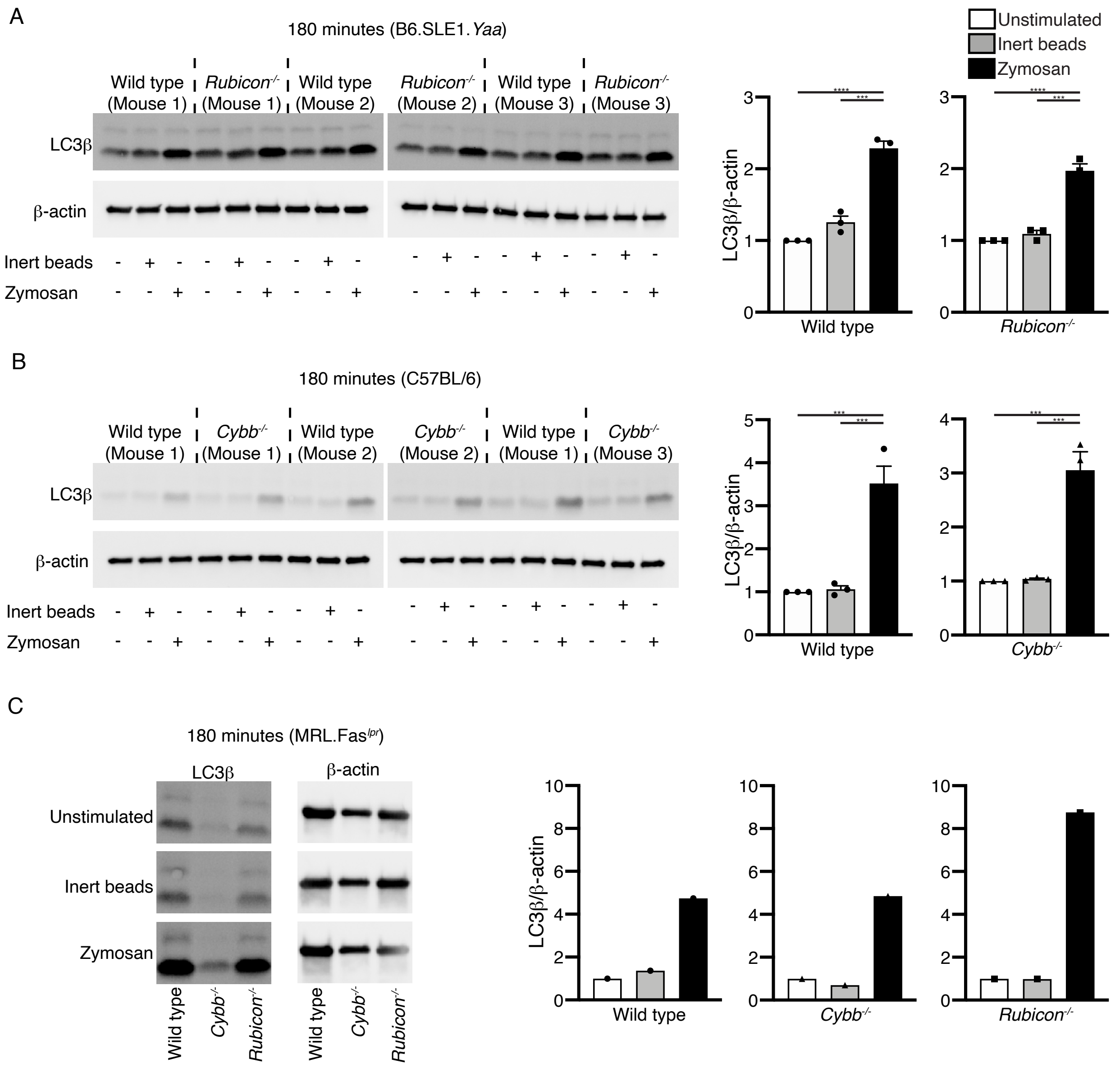

Supplemental Figure 12. *Rubicon* and *Cybb*-deficient bone marrow derived macrophages can undergo LC3-assocated phagocytosis at 180 minutes post-stimulation. (A and B) Bone marrow derived macrophages (BMDMs) generated from (A) *Rubicon*- sufficient (n=3) and -deficient (n=3) B6.Sle1.*Yaa* or (B) *Cybb*- sufficient (n=3) and -deficient (n=3) C57BL/6 mice were left untreated, stimulated with inert polystyrene-BSA beads (8 beads: 1 cell), or stimulated with zymosan bioparticles (8 particles: 1 cell) for 180 minutes. LC3β–I (top band) and LC3β–II (bottom band) were analyzed by immunoblot. **(C)** BMDMs generated from 6-8-week-old wild-type, *Cybb*$^{-/-}$, and *Rubicon*$^{-/-}$ MRL.Fas^{lpr} mice were stimulated as in A. LC3β–II bands were quantitated as described in Figure 6.

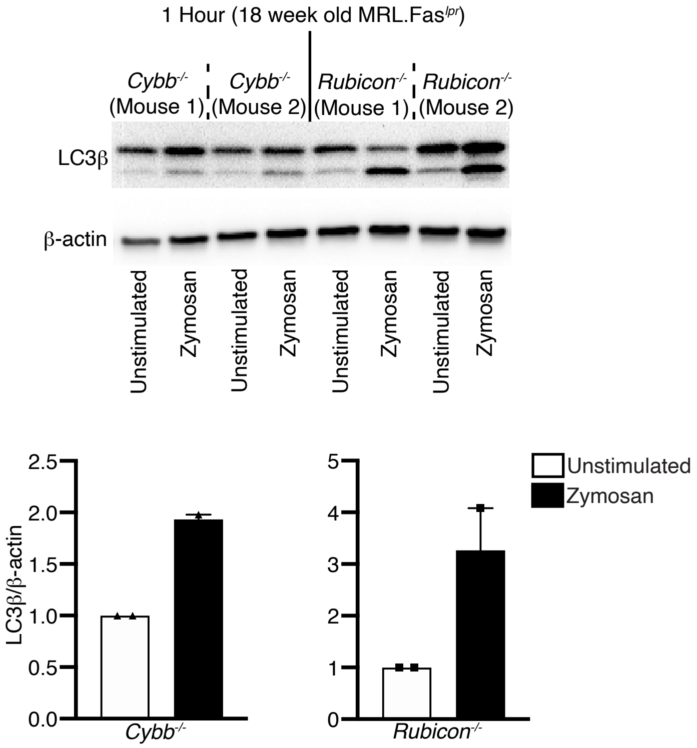

Supplemental Figure 13. Peritoneal macrophages from diseased *Rubicon*$^{-/-}$ and *Cybb*$^{-/-}$ MRL.Fas^{lpr} mice can undergo LC3-assocated phagocytosis at 60 minutes post-stimulation. Peritoneal macrophages isolated from 18-week-old *Cybb*$^{-/-}$ (n=2), and *Rubicon*$^{-/-}$ (n=2) MRL.Fas^{lpr} were left untreated or stimulated with zymosan bioparticles (8 particles: 1 cell) for 60 minutes. LC3β–I (top band) and LC3β–II (bottom band) were analyzed by immunoblot. LC3β–II bands were quantitated as described in Figure 6.

Supplemental Table 1: Percent ± SEM cell composition of male MRL.Fas^{lpr} spleens from indicated strains.

**Cell type**	Wild-type	n	*Rubicon*$^{-/-}$	n	*Cybb*$^{-/y}$	n	*Rubicon*$^{-/-}$*Cybb*$^{-/y}$	n
Neutrophils	3.2±0.3	20	4.1±0.4	19	5.3±0.7	10	5.1±0.4	14
Macrophages	4.3±0.3	20	3.7±0.2	19	4.4±0.5	10	5.3±0.3	14
cDCs	0.4±0.0	20	0.4±0.0	19	0.5±0.1	10	0.4±0.1	14
pDCs	0.1±0.0	20	0.2±0.0	19	0.2±0.0	10	0.2±0.1	14
B cells	37.7±2.6	20	38.5±2.1	19	33.5±4.0	10	38.3±2.3	14
Plasmablasts	1.7±0.2	20	1.36±0.1	19	2.3±0.3	10	1.6±0.2	14
Marginal zone B cells	14.4±1.4	20	13.6±1.0	19	10.6±1.5	10	13.1±1.4	14
Follicular B cells	12.0±1.5	20	12.1±1.3	19	8.7±1.7	10	13.6±1.9	14
Total T cells	32.5±2.3	20	33.8±1.9	19	31.6±3.7	10	32.4±2.1	14
CD4 T cells	11.7±0.8	20	14.2±1.2	19	12.1±1.0	10	12.3±1.1	14
Activated CD4 T cells (% of CD4)	69.3±2.2	20	71.3±1.0	19	67.2±3.7	10	69.4±1.3	14
CD8 T cells	8.5±0.5	20	8.9±0.5	19	7.3±0.4	10	8.7±0.7	14
Activated CD8 T cells (% of CD8)	33.9±1.8	20	30.4±1.6	19	33.5±2.27	10	32±2.7	14

Grey comparisons indicate statistically significant differences (p<0.05) by Student's t test.

Supplemental Table 2: Percent ± SEM cell composition of female MRL.Fas^{lpr} spleens from indicated strains.

**Cell type**	Wild-type	n	*Rubicon*$^{-/-}$	n	*Cybb*$^{-/-}$	n	*Rubicon*$^{-/-}$*Cybb*$^{-/-}$	n
Neutrophils	5.1±0.7	8	4.3±0.5	18	8.0±1.4	5	6.0±0.5	9
Macrophages	4.3±0.6*	8	3.3±0.3	18	7.6±1.4*	5	4.6±0.3	9
cDCs	0.4±0.1	8	0.2±0.0	18	0.3±0.0	5	0.3±0.0	9
pDCs	0.2±0.0	8	0.1±0.0	18	0.2±0.0	5	0.2±0.0	9
B cells	24.9±3.0	8	28.9±2.2	18	25.5±4.5	5	28.5±3.2	9
Plasmablasts	2.8±0.4	8	1.28±0.2	18	3.6±0.6	5	2.9±0.4	9
Marginal zone B cells	7.4±1.6	8	13.3±1.7	18	8.0±1.4	5	9.0±1.2	9
Follicular B cells	5.8±1.0	8	8.9±1.8	18	5.1±1.5	5	6.6±1.1	9
Total T cells	32.3±3.2	8	32.6±2.5	18	37.3±5.6	5	29.5±3.1	9
CD4 T cells	11.8±1.3	8	11.7±0.9	18	15.2±1.0	5	10.0±1.2	9
Activated CD4 T cells	72.9±1.9	8	72.2±2.3	18	69.8±4.1	5	68.0±1.5	9
CD8 T cells	6.9±0.9	8	7.0±0.4	18	8.2±1.5	5	6.1±0.7	9
Activated CD8 T cells	45.54±4.526	8	37.6±2.8	18	34.3±4.9	5	37.5±2.6	9

Grey comparisons indicate statistically significant differences (p<0.05) by Student's t test
* Comparisons indicate statistically significant differences (p<0.05) by Student's t test and One-Way ANOVA with post-hoc Tukey's multiple comparison test.

Supplemental Table 3: Percent ± SEM cell composition of male B6.Sle1.*Yaa* spleens from indicated strains.

**Cell type**	Wild-type	n	*Rubicon*$^{-/-}$	n
Total T cells	17.1±1.8	5	27.6±1.9	10
CD4 T cells	9.9±2.1	5	13±0.7	10
CD44^{+} CD4 T cells	76.4±2	5	63.6±3.6	10
CD8 T cells	4.6±0.8	5	10.35±1.5	10
Tfh CD4 T cells	1.6±0.6	5	3.6±0.6	10

Grey comparisons indicate statistically significant differences (p<0.05) by Student's t test.

Supplemental Table 4: IL-10 deletion efficiency.

**Strain**	Cell Type	n	Mean % deletion efficiency ± SEM	Assay
IL-$10^{fl/fl}$ LysM $Cre^{-/+}$	Neutrophils	22	77.7±0.7	qPCR
	Macrophages	22	43.5±2.6	
	B cells	12	-4.7±7.6	

Supplemental Table 5: Percent ± SEM cell composition of female MRL.Fas^{lpr} spleens from indicated strains.

Cell type	IL-10$^{fl/fl}$ LysM Cre$^{-/-}$ (Male)	n	IL-10$^{fl/fl}$ LysM Cre$^{-/+}$ (Male)	n	IL-10$^{fl/fl}$ LysM Cre$^{-/-}$ (Female)	n	IL-10$^{fl/fl}$ LysM Cre$^{-/+}$ (Female)	n
Neutrophils	2.9±0.2	22	3.1±0.2	31	4.2±0.4	25	3.6±0.6	13
Macrophages	3.2±0.3	22	3.7±0.3	31	3.9±0.4	25	3.1±0.4	13
cDCs	0.2±0.0	22	0.2±0.0	31	0.2±0.0	25	0.3±0.0	13
pDCs	0.1±0.0	22	0.1±0.0	31	0.1±0.0	25	0.1±0.0	13
B cells	29.5±2.7	22	26.3±2.3	31	26.8±2.2	25	19.9±2.8	13
Plasmablasts	1.6±0.2	22	1.7±0.1	31	3.0±0.6	25	2.3±0.4	13
Marginal zone B cells	9.8±1.2	22	9.0±1.2	31	10.2±1.3	25	7.3±1.5	13
Follicular B cells	8.2±1.0	22	10±1.2	31	8.9±1.2	25	5.7±1.0	13
Total T cells	36.7±1.9	22	40.3±2.3	31	38.4±2.0	25	42.0±3.5	13
CD4 T cells	10.0±0.6	22	10.9±0.6	31	11.5±0.5	25	11.9±0.8	13
Activated CD4 T cells	66.8±1.5	22	65.7±1.8	31	66.5±1.1	25	65.6±2.0	13
CD8 T cells	7.5±0.4	22	7.7±0.5	31	6.8±0.5	25	7.3±0.9	13
Activated CD8 T cells	25.3±1.7	22	27.2±1.5	31	31.1±2.0	25	32.50±1.9	13

Grey comparisons indicate statistically significant differences ($p<0.05$) by Student's t test.

Supplemental Table 6: FACS antibodies used for the MRL.Fas^{lpr} cohort analysis.

Antibody	Fluorescence	Clone	Company
IA/E	PE	M5/114.15.2	Biolegend
IA/E	APC/Cy7	M5/114.15.2	Biolegend
Bst-2	Biotin	927	In-house conjugated
CD11c	PE/Cy7	HL3	BD Pharmingen
CD11c	AI488	N418	eBioscience
CD45R	APC/Cy7	RA3-6B2	BD Pharmingen
SiglecH	Al647	eBio440c	Bioscience
Ly6G	Al488	1A8	In-house conjugated
Ly6G	Biotin	1A8	In-house conjugated
Gr1	PE/Cy7	RB6-8C5	Biolegend
Gr1	PE	RB6-8C5	Biolegend
CD11b	APC/Cy7	M1/70	Biolegend
CD11b	PE	M1/70	Biolegend
F4/80	Al647	BM8	In-house conjugated
F4/80	APC	BM8	Biolegend
CD44	Al488	1M7	In-house conjugated
CD44	APC/Cy7	1M7	Biolegend
TcRβ	APC/Cy7	H57-597	Biolegend
TcRβ	PE/Cy7	H57-597	Biolegend
CD62L	PE/Cy7	Mel-14	Biolegend
CD8	Al647	TIB 105	In-house conjugated
CD4	PE	GK1.5	In-house conjugated
CD138	PE	281-2	BD Pharmingen
CD19	Pacblue	1D3.2	In-house conjugated
CD19	Al647	1D3.2	In-house conjugated
Kappa	Pacblue	187.1	In-house conjugated
CD21/CD35	AI488	7G6	In-house conjugated
CD23	PE/Cy7	B3B4	BD Pharmingen
CD19	Al647	1D3.2	In-house conjugated
IgM	Pacblue	B7-6	In-house conjugated
CD93	PE	AA4.1	Biolegend

Supplemental Table 7: FACS antibodies used for the B6.Sle1.*Yaa* cohort analysis.

Antibody	Fluorescence	Clone	Company
IgG	FITC		Southern Biotech
TACI	PE	8F10	BioLegend
CD3	ECD	17A2	BioLegend
CXCR4	PC5.5	L276F12	BioLegend
CD95	PECY7	Jo2	BD
IgM	AF647		BD
CD24	AF700	M1/69	BioLegend
T and B Cell Activation Antigen	BV421	GL7	BD
LD	AmCyan		Invitrogen
CD21	BV605	7G6	BD
IgD	BV650	11-26c.2a	BioLegend
CD138	BV711	281-2	BD
CD86	BV786	GL-1	BioLegend
CD38	BUV395	90/CD38	BD
CD23	BUV737	B3B4	BD
CD16/32	-	2.4G2	BD
CXCR5	Biotin		BD
SA	PE		BD
CCR7	PC5.5	4B12	BD
PD1	PECY7	29F.1A12	BioLegend
BCL6	AF647	K112-91	BD
CD11b	AF700	M1/70	BD
CD11b	BV421	M1/70	BD
PSGL1	BV421	2PH1	BD
CD4	BV650	RM4-5	BioLegend
CD44	BV786	IM7	BioLegend
Ly6C	FITC	AL-21	BD
CD80	PE	16-10A1	BD
CD14	PC5.5	Sa2-B	Invitrogen
CD8a	PECY7	53-6.7	Invitrogen
CD8a	FITC	53-6.7	BioLegend
Ly6G	AF647	1A8	BD
CD11c	AF700	HL3	BD
